# Corrupted coordination of epigenetic modifications leads to diverging chromatin states and transcriptional heterogeneity in CLL

**DOI:** 10.1038/s41467-019-09645-5

**Published:** 2019-04-23

**Authors:** Alessandro Pastore, Federico Gaiti, Sydney X. Lu, Ryan M. Brand, Scott Kulm, Ronan Chaligne, Hongcang Gu, Kevin Y. Huang, Elena K. Stamenova, Wendy Béguelin, Yanwen Jiang, Rafael C. Schulman, Kyu-Tae Kim, Alicia Alonso, John N. Allan, Richard R. Furman, Andreas Gnirke, Catherine J. Wu, Ari M. Melnick, Alexander Meissner, Bradley E. Bernstein, Omar Abdel-Wahab, Dan A. Landau

**Affiliations:** 1 Human Oncology and Pathogenesis Program, Memorial Sloan Kettering Cancer Center, New York, 10065 NY USA; 2 New York Genome Center, New York, 10013 NY USA; 3 Weill Cornell Medicine, New York, 10021 NY USA; 4 Institute for Computational Biomedicine, Weill Cornell Medicine, New York, 10021 NY USA; 5 Broad Institute of MIT and Harvard, Cambridge, 02142 MA USA; 6 Dana-Farber Cancer Institute, Boston, 02215 MA USA; 7 Max Planck Institute for Molecular Genetics, Berlin, 14195 Germany; 8 Department of Pathology, Massachusetts General Hospital, Boston, 02114 MA USA; 9 Leukemia Service, Department of Medicine, Memorial Sloan Kettering Cancer Center, New York, 10065 NY USA

**Keywords:** Chronic lymphocytic leukaemia, Cancer epigenetics, Epigenomics, Gene regulation

## Abstract

Cancer evolution is fueled by epigenetic as well as genetic diversity. In chronic lymphocytic leukemia (CLL), intra-tumoral DNA methylation (DNAme) heterogeneity empowers evolution. Here, to comprehensively study the epigenetic dimension of cancer evolution, we integrate DNAme analysis with histone modification mapping and single cell analyses of RNA expression and DNAme in 22 primary CLL and 13 healthy donor B lymphocyte samples. Our data reveal corrupted coherence across different layers of the CLL epigenome. This manifests in decreased mutual information across epigenetic modifications and gene expression attributed to cell-to-cell heterogeneity. Disrupted epigenetic-transcriptional coordination in CLL is also reflected in the dysregulation of the transcriptional output as a function of the combinatorial chromatin states, including incomplete Polycomb-mediated gene silencing. Notably, we observe unexpected co-mapping of typically mutually exclusive activating and repressing histone modifications, suggestive of intra-tumoral epigenetic diversity. Thus, CLL epigenetic diversification leads to decreased coordination across layers of epigenetic information, likely reflecting an admixture of cells with diverging cellular identities.

## Abstract (web summary)

In chronic lymphocytic leukemia (CLL), evolution is driven by transcriptional and epigenetic heterogeneity. Here, the authors integrate epigenomic analyses to show how intra-tumoral epigenetic diversity results in divergent chromatin states in CLL cells, increasing cell-to-cell transcriptional heterogeneity.

## Introduction

Cancer growth, progression, and relapse are the result of an evolutionary process fueled by intra-tumoral diversity^1–3^. Chronic lymphocytic leukemia (CLL)—a common B cell malignancy—serves as a highly informative model for cancer evolution as it undergoes substantial genetic diversification^4^ and evolution with therapy^5^.

In addition to genetic changes, the CLL epigenome is an important disease-defining feature linked to its cell-of-origin and is predictive of outcome^6–8^. In fact, the stable propagation of the ancestral epigenome allowed the use of DNA methylation (DNAme) patterns to precisely retrace the initially transformed cell-of-origin from which different CLLs emerge^8^. In addition to the largely stably inherited epigenome, we have previously shown that growing CLL populations also undergo ongoing somatic DNAme changes akin to the process of genetic diversification through ongoing mutations, leading to high intra-leukemic epigenetic heterogeneity, greater clonal evolution, and adverse outcome^9^, as has been shown for other malignancies^10^.

However, DNAme constitutes only a single layer of the epigenetic information encoding cell identity. Given the importance of histone modifications to lineage plasticity in cancer^11,12^, we reasoned that intra-leukemic epigenetic heterogeneity may extend to histone modifications, likely promoting lineage plasticity by enabling permissive chromatin states. To address this question, we complemented bulk reduced representation bisulfite sequencing (RRBS) analysis with a chromatin immunoprecipitation sequencing (ChIP-seq) compendium of histone post-translational modifications and gene expression, together with joint DNAme and transcriptome single cell analysis in a cohort of 22 primary CLL and 13 healthy B lymphocytes samples. Our integrative analysis revealed a markedly decreased coordination between different layers of the CLL epigenome, whereby ongoing epigenetic diversification leads to an admixture of cells with diverging epigenetic identities, thus providing a novel perspective into the epigenetic dimension of cancer evolution.

## Results

### Super-enhancer and associated DNAme alteration in CLL

To comprehensively study the epigenetic landscape of evolving CLL and its relationship to intra-leukemic diversity, we generated genome-wide maps of histone marks with non-overlapping regulatory functions (H3K4me3, H3K27ac, and H3K27me3) and transcriptome sequencing (bulk RNA-seq) in a cohort of 20 primary *IGHV* mutated and unmutated CLL (corresponding to the major known disease subtypes^13^; *n* = 14 and *n* = 6, respectively), as well as 12 healthy B lymphocytes samples (CD19/CD23/IgD-positive CD27-negative tonsillar naïve B cells [NBCs; CD19^+^CD23^+^CD27^−^IgD^+^], *n* = 2; peripheral blood NBCs [CD19^+^CD23^+^CD27^−^IgD^+^], *n* = 4; CD19/CD23/CD27-positive IgD-negative tonsillar germinal center B cells [GCBs; CD19^+^CD23^+^CD27^+^IgD^−^], *n* = 2; peripheral blood memory B cells [GCBs; CD19^+^CD23^+^CD27^+^IgD^−^], *n* = 3; CD20-positive tonsillar B cells [CD20^+^], *n* = 1; Supplementary Fig. 1a, b).

**Fig. 1:**
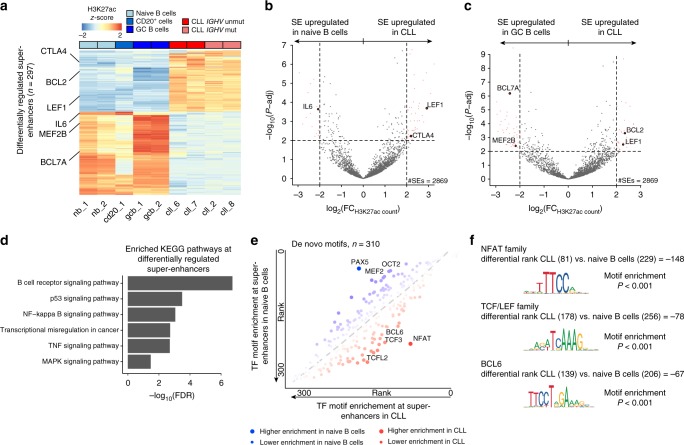
Super-enhancer rewiring in CLL. **a** H3K27ac profiles of 297 differentially regulated super-enhancers (absolute log_2_[H3K27ac fold-change] > 2 and Wald test BH-FDR < 0.01) between CLL and normal B cells. Red indicates high H3K27ac level, blue low H3K27ac level. Gene assignment to super-enhancers based on proximity (average distance of 2775 bp). Genes critical for lymphocyte proliferation and differentiation are highlighted. **b** Differential H3K27ac at super-enhancers (*n* = 2869) between CLL and naïve B cells (same samples as in **a** were used in the analysis). Differentially regulated super-enhancers defined as having absolute log_2_(H3K27ac fold-change) > 2 and Wald test BH-FDR < 0.01. **c** Same as **b** for differential H3K27ac at super-enhancers (*n* = 2869) between CLL and germinal center B cells. **d** KEGG pathways enriched at differentially regulated super-enhancers (*n* = 297; hypergeometric test BH-FDR < 0.01). **e** Comparison of rank in transcription factor de novo motif enrichment (*n* = 310, ranked by hypergeometric test *P*-value) between CLL (*x*-axis) and naïve B cells (*y*-axis) at super-enhancers. Critical TF motifs for lymphocyte proliferation and B cell differentiation are highlighted. LOESS regression line of observed ranked *P*-values is shown in dotted gray. Color: red—CLL biased; blue—naïve B cell biased, size adjusted based on residual. **f** Position weight matrices of selected de novo TF motifs significantly over-represented in CLL (standardized residuals > 1.65). For each motif, the differential ranked *P*-value between CLL and naïve B cells, motif enrichment hypergeometric test *P*-value, and the best match to JASPAR core database are shown

Analysis of H3K27ac, a histone modification known to be a marker of active gene regulatory regions^14^, revealed core enhancer and super-enhancer (as defined in ref. ^14^; see Methods; Supplementary Fig. 1c) reprogramming in CLL. A total of 297 super-enhancers were differentially regulated in CLL compared with normal B cells (absolute log_2_[H3K27ac fold-change] >2 and Wald test BH-FDR <0.01; see Methods), with increased H3K27ac in proximity to genes critical for lymphocyte proliferation and differentiation, including *BCL2*, *LEF1*, and *CTLA4*^15–17^ (Fig. 1a–c; Supplementary Fig. 1d) and involved in pathways previously reported to play key roles in CLL (e.g., B cell receptor, NF-kB and MAPK inflammatory signaling pathways^3^; Fig. 1d). As ChIP-seq experiments are prone to technical variation, we further demonstrated the reproducibility of H3K27ac derangements in CLL by analyzing additional CLL and normal B cell samples from the Blueprint Initiative^18^ (Supplementary Data 1), showing high pairwise correlations across our cohort and the Blueprint initiative samples at super-enhancers (Supplementary Fig. 1e). Fewer differences in the super-enhancer landscape were observed between the two major known CLL subtypes (*IGHV* mutated and unmutated; *n* = 27 super-enhancers differentially regulated; Supplementary Fig. 1f; see Methods), and with chromosome 13q deletion (del(13)q; *n* = 25 super-enhancers differentially regulated; Supplementary Fig. 1g; see Methods), consistent with previous studies showing more subtle chromatin differences between CLL subtypes^19^. In line with prior studies that profiled epigenomic features of a large CLL cohort and discrete normal B cell subtypes along the differentiation program^8,19^, this extensive chromatin rewiring at super-enhancers is mediated by specific transcription factors, as evidenced by enrichment of their motifs in activated super-enhancers, including *NFAT*, a deregulated gene with functional and therapeutic potential in CLL^8^, and *TCFL2*, a downstream target of the WNT pathway overexpressed in CLL^20^ (Fig. 1e, f; Supplementary Data 2, 3).

DNAme changes at enhancers and super-enhancers impact their transcriptional activity^21^. Therefore, to assess the relationship between DNAme and enhancers, we profiled bulk DNAme of normal B cell populations (peripheral blood naïve B cells [CD19^+^CD23^+^CD27^−^IgD^+^], *n* = 3; peripheral blood memory B cells [CD19^+^CD23^+^CD27^+^IgD^−^], *n* = 2) and CLL patient samples (*IGHV* unmutated, *n* = 2; *IGHV* mutated, *n* = 3) using a targeted bisulfite sequencing capture assay, which preferentially evaluates dynamic CpGs at gene-regulatory elements^22^ (Supplementary Fig. 1a, b; Supplementary Fig. 2a, b; Supplementary Data 4, 5). Consistent with prior reports^7,9,23^, we observed a global decrease in DNAme in CLL compared with normal B samples (Supplementary Fig. 2c, left; Supplementary Fig. 2d), with a focal increase in methylation of CpG islands (CGI; Supplementary Fig. 2c, right).

**Fig. 2:**
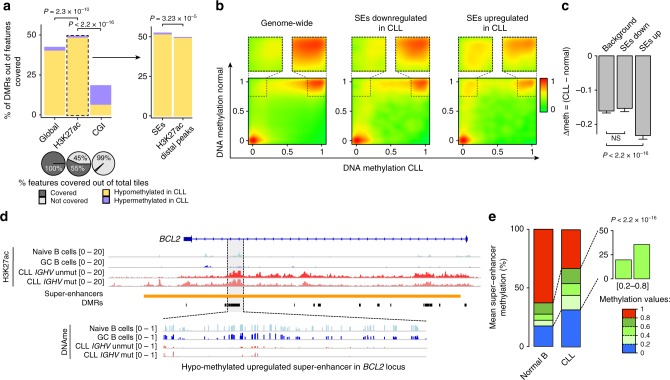
DNA methylation alteration at super-enhancers in CLL. **a** Percentage of differentially methylated regions (DMRs) measured with targeted bisulfite sequencing capture assay in (i) Global: all covered 500bp-tiles in the genome; (ii) H3K27ac: union of all H3K27ac peaks; (iii) CGI: CpG islands; (iv) SE: union of all super-enhancers; (v) H3K27ac distal peaks: union of all H3K27ac peaks that did not overlap TSSs (±2.5 kb). *P*-values are shown for two-sided Fisher’s exact test. Bottom: percentage of distinct genomic features covered by the targeted bisulfite sequencing capture assay. **b** CpG methylation for CLL and normal B cells across all super-enhancers (left), super-enhancers upregulated in normal B cells (center), and super-enhancers upregulated in CLL (right), measured with targeted bisulfite sequencing capture assay. **c** Difference in mean CpG methylation between CLL and normal B cells for the three categories shown in **b**. Error bars represent 95% confidence interval. *P-*values are indicated for two-sided Mann–Whitney *U*-test. NS, not significant. **d** Epigenomic profiling of the *BLC2* locus in CLL compared with normal B cells. **e** The percentage of CpG methylation values at super-enhancers in CLL (no. of CpGs used = 468,303) and normal B cells (no. of CpGs used = 502,607), measured with targeted bisulfite sequencing capture assay. *P*-value is shown for two-sided Fisher’s exact test for the intermediate category [0.2–0.8]

In addition, we identified 41,057 differentially methylated regions (DMRs; absolute change in DNAme > 0.3 and Fisher’s exact test FDR <0.05^22^; see Methods) between CLL and normal B samples, most of which were hypomethylated in CLL (Supplementary Fig. 2e; Supplementary Data 6–8). Interestingly, hypomethylation preferentially affected H3K27ac-enriched regions, including super-enhancers (Fisher’s exact test *P* < 0.0001; Fig. 2a; Supplementary Fig. 2e, f). This extensive focal hypomethylation at super-enhancers was observed in proximity to genes involved in pathways previously reported to play key roles in CLL (e.g., B cell receptor activation, Notch signaling, and cell proliferation^3^; Supplementary Fig. 2g; Supplementary Data 9). Additionally, CLL-specific super-enhancers showed a strong decrease in DNAme compared to normal B samples (Mann–Whitney *U*-test, *P* < 0.0001; Fig. 2b, c), as illustrated for the *BCL2* gene locus (Fig. 2d). In contrast, super-enhancers that become inactive in CLL did not gain DNAme compared to normal B samples (Mann–Whitney *U*-test*, P* > 0.05; Fig. 2b–d), supporting the concept that DNAme is slow to accumulate with CLL progression^19,24^.

Notably, we observed that hypomethylation at super-enhancers resulted preferentially in intermediate DNAme levels in CLL (Fisher’s exact test *P* < 0.0001; Fig. 2e; Supplementary Fig. 2h). These data demonstrated that cancer-associated hypomethylation is not limited to previously described intermediately methylated blocks in heterochromatin and lamina associated domains^25,26^, but may also involve regions of active chromatin.

### Decreased epigenetic-transcriptional coordination in CLL

**Fig. 3:**
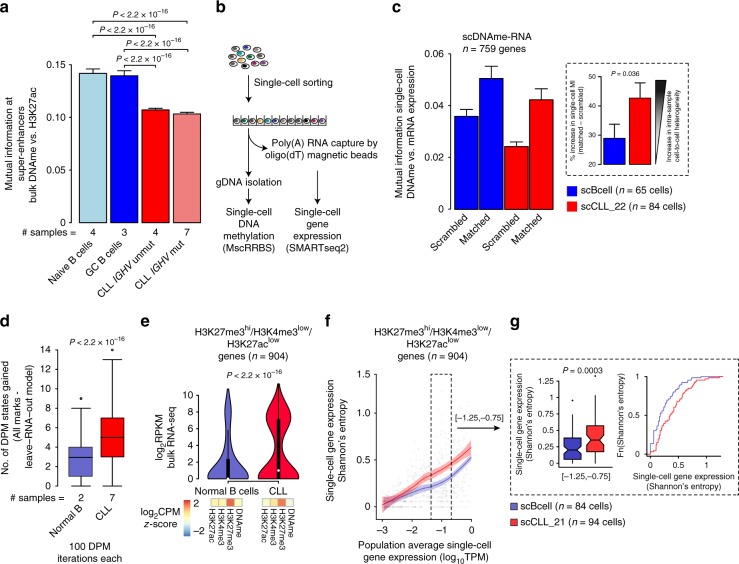
Decreased epigenetic-transcriptional coordination in CLL. **a** Mutual information (MI) across super-enhancers (*n* = 2869) between bulk DNAme (measured with targeted bisulfite sequencing capture assay) and H3K27ac for CLL and normal B samples. *P-*values are indicated for two-sided Welch’s *t*-test. **b** Schematic of the experimental procedures of the joint multiplexed single-cell RRBS and transcriptomics analysis. **c** MI between expression and promoter single-cell DNAme rate (*n* = 759 genes with sufficient RNA [expression seen in >5 cells] and DNAme [>10 CpGs per promoter] information) in individual CLL cells (*n* = 84) and normal B cells (*n* = 65). The observed MI values were compared to values obtained by randomly permuting cell labels for the methylation values. Inset: percentage increase in MI when comparing matched vs. scrambled single-cell DNAme and RNA data in CLL and normal B cells. *P*-value is indicated for two-sided Mann–Whitney *U*-test. **d** Number of states gained when adding RNA data to the epigenomic mapping (H3K4m3, H3K27ac, H3K27me3, DNAme based on bulk bisulfite sequencing) in the DPM analysis for CLL and normal B samples. *P*-value is indicated for two-sided Mann–Whitney *U*-test. See also Supplementary Fig. 3d. **e** Expression levels (log_2_[RPKM]) of 904 genes marked by H3K27me3^hi^/H3K4me3^low^/H3K27ac^low^ between normal B samples (blue) and CLL (red) samples (*n* = 2 and *n* = 7, respectively). *P*-value is shown for two-sided Mann–Whitney *U*-test. **f** Single-cell gene expression Shannon’s information entropy in relation to the population average gene expression (based on single-cell whole transcriptome data) in CLL (*n* = 94) and normal B cells (*n* = 84). Local regression lines for the H3K27me3^hi^/H3K4me3^low^/H3K27ac^low^-marked genes in CLL (red) and normal B cells (blue) are shown. **g** Single-cell gene expression Shannon’s information entropy (left) for genes within a population average expression range of −1.25 to −0.75 log_10_(TPM) (to control for differences in this variable). *P*-values are shown for two-sided Mann–Whitney *U*-test. Cumulative distribution (right) showing the proportion of intermediate single-cell gene expression Shannon’s information entropy values at H3K27me3^hi^/H3K4me3^low^/H3K27ac^low^-marked genes. Throughout the figure: boxplot represents median and bottom and upper quartile; whiskers correspond to 1.5*IQR; error bars represent 95% confidence interval

The observed intermediate bulk DNAme patterns at H3K27ac regulatory regions are reminiscent of our previous observation of intermediate DNAme in promoters stemming from stochastic DNAme intra-leukemic diversification during CLL evolution^9^. Therefore, to examine whether enhancer rewiring is also associated with disordered methylation leading to reduced coordination between DNAme and H3K27ac, we drew on a well-established metric in information theory—mutual information (MI)—which measures how much can be learned from one variable about another (see Methods). Consistent with disrupted coordination across these two layers of the CLL epigenome, we observed lower pairwise MI between bulk DNAme and H3K27ac in CLL samples (irrespective of their *IGHV* mutational status) compared with normal B cell samples at super-enhancer regions (Welch’s *t*-test*, P* < 0.0001; Fig. 3a; Supplementary Fig. 3a). This decrease in MI also corresponded to a weaker correlation between DNAme and H3K27ac in CLL samples compared with normal B cell samples at super-enhancers (linear regression *R*^2^ of 0.558 normal B vs. 0.471 CLL samples, *t*-test *P* < 0.0001).

The decrease in MI was observed more broadly, including a 13% decrease in MI between DNAme at transcription start sites (TSSs) and gene expression, in CLL relative to normal B cells (Supplementary Fig. 3b). This decrease in MI may result from greater intra-leukemic cell-to-cell heterogeneity that is not captured in bulk population sequencing assays. To directly test this hypothesis, we performed joint single-cell DNAme sequencing and whole transcriptome sequencing on additional normal B and CLL samples (*n* = 96 cells [1 sample], *n* = 288 cells [2 samples], respectively; Fig. 3b; Supplementary Fig. 3c). While MI was higher across samples in matched vs. scrambled single-cell DNAme and RNA-seq data (paired *t*-test, *P* < 0.0001), the matched single-cell MI increase was higher in CLL compared with normal B cells (43 ± 5.2% vs. 29 ± 4.8%, respectively; Mann–Whitney *U*-test, *P* = 0.036; Fig. 3c). These data suggest that, at least in part, the decreased epigenetic-transcriptional coordination observed in CLL is the result of cell-to-cell epigenetic diversification.

To more broadly examine the relationship between epigenetic states (i.e., combinatorial interactions of epigenetic marks) and transcriptional output, we modeled the combinatorial patterns of histone modifications (H3K4me3, H3K27ac, H3K27me3) and DNAme (based on bulk bisulfite sequencing), with or without gene expression (based on bulk RNA-seq), using a Dirichlet Process Mixture (DPM) approach, which allows learning de novo the number of combinatorial states. We observed a significantly higher number of states across CLL samples (in both *IGHV* mutated and unmutated samples), compared with normal B cells when adding RNA information into the DPM analysis, indicating that the transcriptional output of epigenetic states is less uniform in CLL (Mann–Whitney *U*-test, *P* < 0.0001; Fig. 3d; Supplementary Fig. 3d). Specifically, while H3K27me3^hi^/H3K4me3^low^/H3K27ac^low^-marked genes (*n* = 904) were associated with uniform gene silencing in B cells, they were associated with variable expression in CLL (Mann–Whitney *U*-test*, P* < 0.0001; Fig. 3e), suggesting that disrupted Polycomb repression in CLL results in leaky silencing allowing partial reactivation of these genes. Notably, variable expression of H3K27me3^hi^/H3K4me3^low^/H3K27ac^low^-marked genes preferentially affected genes related to the critical B-cell receptor (BCR) signaling pathway (Supplementary Fig. 3e). In addition, we observed enrichment of specific transcription factor binding motifs in H3K27me3^hi^/H3K4me3^low^/H3K27ac^low^-marked regions, including *NFAT*^8^ and *MYB*, a proto-oncogene overexpressed in CLL^27^ (Hypergeometric test *P* < 0.0001; Supplementary Fig. 3f). These data suggest that CLL epigenomes are associated with less uniform transcriptional outputs compared with normal B cell epigenomes.

Transcriptional variation in genes with similar epigenetic states may stem from cell-to-cell transcriptional heterogeneity. To test this, we computed gene expression information entropy, a measure of cell-to-cell gene expression heterogeneity^9^ in our single-cell whole transcriptome data, and found that in CLL single cells (*n* = 94) H3K27me3^hi^/H3K4me3^low^/H3K27ac^low^-marked genes were indeed associated with significantly higher intra-leukemic expression information entropy compared to normal B cells (*n* = 84), or compared to a set of genes with matched mean expression but not marked by H3K27me3^hi^/H3K4me3^low^/H3K27ac^low^ (Mann–Whitney *U*-test, *P* = 0.0003 and *P* = 0.005, respectively; Fig. 3f, g; Supplementary Fig. 3g). Our data therefore suggest a model in which H3K27me3-marked genes in CLL are incompletely silenced, resulting in greater cell-to-cell transcriptional heterogeneity.

### Corrupted coherence across layers of the CLL epigenome

**Fig. 4:**
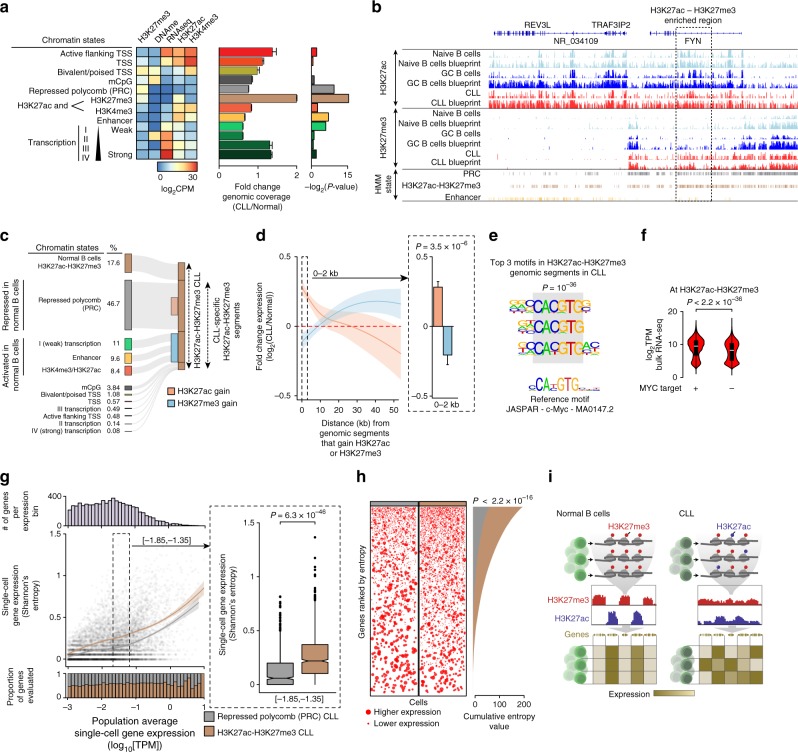
Corrupted coherence across layers of CLL epigenome leads to cell-to-cell transcriptional heterogeneity. **a** Chromatin state definitions and enrichments for a 12-state Hidden Markov Model based on three histone marks (H3K4me3, H3K27ac, H3K27me3), DNAme, and RNA information. *P*-values of a given HMM state between CLL and normal B cells are shown for two-sided hypergeometric test. **b** Epigenomic profiling of the *FYN* gene locus, demonstrating “H3K27a-H3K27me3” state increase in CLL compared with normal B cells across our cohort and Blueprint initiative samples. **c** Sankey diagram showing that ~47% of the regions in a “H3K27ac-H3K27me3” state in CLL carried repressive chromatin modifications in B cells. **d** Fold-change gene expression between CLL and normal B cells in relation to genomic distance from regions that gain H3K27ac (orange; *n* = 11,740 genes) or H3K27me3 (blue; *n* = 8867 genes) in CLL. Mann–Whitney *U*-test. **e** Position weight matrices of the top three motifs over-represented in CLL “H3K27ac-H3K27me3” regions. Motif enrichment hypergeometric test *P*-value and the best reference motif match (JASPAR core database) are shown. **f** Expression levels (log_2_[TPM]) of *MYC* target genes (containing promoter *MYC* binding motif, as in analysis in **e**) compared with non-*MYC* target genes in “H3K27ac-H3K27me3” regions in CLL. Mann–Whitney *U*-test. **g** Single-cell (*n* = 94) gene expression Shannon’s information entropy in relation to the population average gene expression in CLL (scCLL_21). Colored lines—local regression curves for genes in a ‘H3K27ac-H3K27me3’ (brown) or “Repressed Polycomb (PRC)” (gray) state. Inset: single-cell gene expression Shannon’s information entropy for each of the two chromatin states comparing genes within a defined range of population average gene expression. Mann–Whitney *U*-test. **h** Gene expression magnitudes of genes in “Repressed Polycomb (PRC)” state and genes in “H3K27ac-H3K27me3” state. Cumulative distribution (right) showing the proportion of intermediate single-cell gene expression Shannon’s information entropy values at these genes is also shown. Kolmogorov–Smirnov test. **i** Well-coordinated chromatin programs stabilize gene expression and cellular identities in normal B cells (left). On the contrary, intra-leukemic epigenetic diversity results in a permissive chromatin state in CLL cells (right), enhancing cell-to-cell transcriptional variation. Boxplots represent median and bottom and upper quartile; whiskers correspond to 1.5*IQR; error bars represent 95% confidence interval

An alternative approach to assess the coordination between layers of the epigenome involves capturing their overlapping and mutually exclusive combinatorial patterns^28^. We pursued this orthogonal approach by training a multivariate Hidden Markov Model (HMM) on CLL and normal B cells data based on three of the different histone modifications (H3K4me3, H3K27ac, H3K27me3), DNAme (based on bulk bisulfite sequencing), and gene expression information (based on bulk RNA-seq). We identified 12 distinct epigenetic states that fell into two broad categories. First, a category that correlated with active transcription including active promoters (“Active flanking TSS”, “TSS”), enhancers (“Enhancer”, “H3K4me3/H3K27ac”), and 5′ and 3′ boundaries of transcribed genes (“I–IV transcription”). Second, a category of genes with no or little detectable transcription, including bivalent or poised (“Bivalent/Poised TSS”), repressed Polycomb (“PRC”), and mCpG-rich (“mCpG”) states (Fig. 4a).

CLL overall showed high resemblance to normal B cells, and no significant differences in genomic coverage were observed between *IGHV* mutated and unmutated CLL (Supplementary Fig. 4a). Importantly, HMM analysis revealed a chromatin state simultaneously marked by H3K27ac and H3K27me3, modifications which are typically mutually exclusive, with a >2-fold enrichment in CLL compared with normal B cells (Hypergeometric test *P* < 0.0001; Fig. 4a, b), and affecting ~1.6 M 200 bp genomic segments including non-first introns and distal regulatory elements (Supplementary Fig. 4b). We further validated this chromatin state by analyzing an additional 6 CLL and 7 normal B cell samples from the Blueprint Initiative^18^ and obtained high pairwise correlation (Spearman’s rho correlation coefficient = 0.47) between H3K27ac and H3K27me3 marks at H3K27ac-H3K27me3 segments identified in our data (Supplementary Fig. 4c). Evaluation of these H3K27ac-H3K27me3 segments from CLL revealed that a notable fraction (46.7%) of these regions possessed repressive chromatin modifications in normal B cells (Fig. 4c), suggesting these are genomic regions that are subject to CLL-specific activation by gaining activating acetylation marks (H3K27ac). Gaining H3K27ac in the transition from a healthy to disease state may be associated with upregulation of neighboring genes. Consistent with this scenario, RNA gene expression was increased in proximity to regions that gain H3K27ac in CLL (Fig. 4d; Supplementary Fig. 4d). A gene set enrichment analysis of closest genes to these regions revealed enrichment in gene sets associated with stem cell identity^29,30^ (Hypergeometric test BH-FDR <0.05), linking regulatory chromatin variability to stem-like cell programs, according to the notion that epigenetic variability in cancer may lead to a drift toward a hybrid stem-somatic cell state^9,31^ (Supplementary Fig. 4e; Supplementary Data 10).

Epigenetic factors, such as aberrant regulation of H3K27 methylation^12^ and sporadic TF activation^32^, have been recently implicated in promoting lineage plasticity in cancer. Thus, to identify which TFs may carry the potential to rewire CLL cells and promote lineage plasticity in CLL, we further mined the regions marked by H3K27ac-H3K27me3 for transcription factor motif enrichment and identified a significant enrichment of the proto-oncogene *MYC* motif, a TF associated with lineage plasticity and CLL transformation to aggressive large B cell lymphoma^33^ (Hypergeometric test *P* < 0.0001; Fig. 4e). RNA gene expression of genes with a *MYC* binding motif at their promoters was increased compared with non-*MYC* target genes, in the regions marked by H3K27ac-H3K27me3 (median [IQR] of 9.44 [4.34] vs. 8.23 [5.17] log_2_[TPM], respectively; Mann–Whitney *U*-test, *P* < 0.0001; Fig. 4f).

Notably, the observed co-mapping of H3K27ac and H3K27me3 to the same genomic locus in CLL may arise from cell-to-cell divergence in histone modification, rather than co-occurrence of these mutually exclusive marks in the same cells. Consistent with this hypothesis, we analyzed our single-cell whole-transcriptome data and observed that genes neighboring H3K27ac-H3K27me3 regions in CLL were associated with higher intra-leukemic expression information entropy in single cells compared with genes neighboring Polycomb repressed regions (Mann–Whitney *U*-test, *P* < 0.0001; Fig. 4g, h; Supplementary Fig. 4f, g). Collectively, these data suggest that CLL cell populations lose effective Polycomb repression of *MYC* targets, likely enabling an exploration of transcriptional stem-like cell programs in CLL evolution.

## Discussion

While cancer evolution investigations have focused on genetic alterations, emerging data across cancer also highlighted the contribution of heritable epigenetic changes to cancer evolution^11,12,32^. In this study, we provided an integrative analysis of the epigenetic landscape of CLL and its relationship to intra-leukemic epigenetic and transcriptional diversity.

We observed extensive chromatin rewiring at H3K27ac regulatory regions mediated by specific transcription factor families, in particular NFAT and TCF/LEF transcription factor families^8,19,20^. Through targeted bisulfite sequencing capture assay, we further showed these regulatory regions to display the highest degree of change in DNAme. Notably, enhancer hypomethylation is preferentially associated with intermediate DNAme levels, likely reflecting intra-leukemic cell-to-cell heterogeneity^9,10^. Thus, intermediately methylated regions in cancer may not be limited to heterochromatin as previously described^25,26^, affecting also regions of regulatory chromatin.

Moreover, while normal B cells exhibit coordinated epigenetic-transcriptional regulation resulting in higher pairwise mutual information, CLL samples have a substantial decrease in DNAme-RNA mutual information. This finding is consistent with intra-leukemic heterogeneity decreasing the mutual information of these two variables when measured at the population level. To directly examine this scenario, we applied matched DNAme and mRNA single-cell information and found a greater increase in single-cell mutual information in CLL compared with normal B cells. This observation confirms that the relatively small contribution of promoter DNAme to explaining transcriptional variation in bulk cancer studies^9^ results, at least in part, from intra-leukemic epigenetic diversity.

To further extend the evaluation of epigenetic co-ordination beyond two epigenetic layers, we modeled the combinatorial patterns of histone modifications, DNAme, and gene expression. Interestingly, we observed a dysregulation of the transcriptional output as a function of the combinatorial chromatin states. Specifically, while in normal B cells H3K27me3^hi^/H3K4me3^low^/H3K27ac^low^-marked genes were generally associated with a uniform transcriptional output, in CLL these genes were associated with variable expression level. As H3K27me3 is typically deposited at gene promoters by Polycomb Repressive Complex 2 (PRC2) via its catalytic Ezh2/Ezh1 subunit^34^, these results are consistent with CLL epigenetic landscape being marked by incomplete Polycomb complex-mediated gene silencing resulting in permissive chromatin states in a fraction of cells. Furthermore, as DNAme is important for appropriate retargeting of PRC2 and H3K27me3 histone modification across cell divisions^35^, stochastic DNAme alterations during CLL evolution^9^ may lead to redistribution of the repressive activity of the PRC2 complex and the H3K27me3 mark, and cell-to-cell variation in the efficiency of PRC2 transcriptional silencing^36^.

Lastly, we observed an unexpected co-occurrence of typically mutually exclusive activating (H3K27ac) and repressing (H3K27me3) histone modifications, closely associated with activation of stem-like programs and greater cell-to-cell transcriptional heterogeneity. Notably, the co-mapping of these typically mutually exclusive histone modifications was previously observed in the context of embryonic stem cell neural differentiation, reflecting cellular heterogeneity due to admixture of differentiated and undifferentiated cells^37^. Thus, epigenetic diversification leads to corrupted coherence across the different layers of the epigenome in CLL, consistent with ongoing epigenetic diversification leading to an admixture of cells with diverging epigenetic identities (Fig. 4i).

While genetic heterogeneity plays a key role in cancer growth, progression, and evolution with therapy^3,5,38,39^, epigenetic evolutionary routes are a major emerging theme across cancer, including prostate cancer, lung cancer and melanoma^11,40^. Cancer cells can display profound non-genetically mediated transcriptional variability, which may enable adaptive changes such as therapeutic resistance, persistence or lineage plasticity. Notably, these states are efficiently propagated to progeny cells suggesting stable epigenetic encoding. Indeed, in CLL, non-genetic persistence as well as lineage transformation have been reported as potential routes of escape from therapeutic inhibition^5,38,41^. Our data demonstrates that these adaptive capacities may be fueled by significant intra-tumoral epigenetic diversity resulting in permissive chromatin states across cells, leading to greater cell-to-cell transcriptional variation (Fig. 4i). Thus, intra-tumoral epigenetic diversity may permit leukemic cells to stochastically activate alternate gene regulatory programs, facilitating the emergence of novel cell states, ultimately fostering CLL’s ability to efficiently explore the fitness landscape for superior evolutionary trajectories during tumorigenesis and in response to therapy.

## Methods

### Human subjects, sample collection, and genotyping

The study was approved by the local ethics committee and by the Institutional Review Board (IRB) and conducted in accordance to the Declaration of Helsinki protocol. Blood samples were collected in EDTA blood collection tubes (BD Biosciences) from patients and healthy adult volunteers enrolled on clinical research protocols at the Dana-Farber/Harvard Cancer Center (DF/HCC), Memorial Sloan Kettering Cancer Center (MSKCC), and NewYork-Presbyterian/Weill Cornell Medical Center (NYP/WCMC). We note that the IRB does not permit collection of demographic information of healthy donors. Informed consent on DF/HCC, MSKCC and WCMC IRB-approved protocols for genomic sequencing of patient samples was obtained prior to the initiation of sequencing studies. The diagnosis of CLL according to World Health Organization (WHO) criteria was confirmed in all cases by flow cytometry, or by lymph node or bone marrow biopsy. B cells from healthy donors and CLL patient samples were isolated from blood samples using Ficoll-Paque Plus (GE Healthcare) density gradient centrifugation and red blood cell lysis, followed by EasySep™ Human B Cell Enrichment Kit (STEMCELL Technologies, Vancouver, Canada) as per manufacturer recommendation. Cells were then cryopreserved in 50% FBS/40% RPMI/10% DMSO and stored in vapor-phase liquid nitrogen until the time of analysis. Tonsillar B cell populations were affinity-purified from de-identified human tonsillectomy specimens by magnetic cell separation^42^, cryopreserved in 50% FBS/40% RPMI/10% DMSO and stored in vapor-phase liquid nitrogen until the time of analysis. White blood cells (WBC) counts for the CLL patient samples used in our analyses were in a range of 50–394 K (median of 201 K), consistent with a purity of >90% based on previously published sequencing data^3^. Immunoglobulin heavy-chain variable (*IGHV*) homology (unmutated was defined as greater than or equal to 98% homology to the closest germline match) were determined^43^. Cytogenetics were primarily evaluated by FISH analysis for the most common CLL abnormalities [del(13q), trisomy 12, del(11q), del(17p), del(6q), amp(2p)] (Supplementary Fig. 1b); if FISH was unavailable, genomic data were used.

### Antibodies

Purified CD19^+^ naive B cells (CD19^+^CD23^+^CD27^−^IgD^+^) and germinal center memory B cells (CD19^+^CD23^+^CD27^+^IgD^−^) were sorted using PE/Cy7 anti-human CD27 (1:5 dilution; clone O323, Bio Legend) and FITC mouse anti-human IgD (1:5 dilution; clone IA6-2, BD Pharmingen) antibodies with a FACSAria II instrument (Becton Dickinson, Franklin Lakes, NJ). Tonsillar CD20^+^ cells were sorted as CD19^+^CD20^+^CD38^++^. CD5^+^ normal B cells were not profiled due to their low frequency, and previous data^7^ showed minimal DNA methylation differences between CD5^+^ and CD5^−^ naïve B cells. Antibodies used for ChIP include anti-H3K4me3 (1 mg for 50 mg of chromatin; 9751S Cell Signaling, Danvers, MA), anti-H3K27ac (2 mg for 25 mg of chromatin; ab4729 Abcam, Cambridge, United Kingdom), anti-H3K27me3 (2 mg for 25 mg of chromatin; 07–449 Millipore, Burlington, MA).

### ChIP-seq and motif analysis

A minimum of 2 million purified human cells were used. Briefly, cells were fixed in a 1% methanol-free formaldehyde solution and then resuspended in sodium dodecyl sulfate (SDS) lysis buffer. Lysates were sonicated in an E220 focused-ultrasonicator (Covaris, Woburn, MA) to a desired fragment size distribution of 100–500 base pairs. ChIP assays were processed on a SX-8G IP-STAR Compact Automated System (Diagenode, Denville, NJ) using a direct ChIP protocol^44^. Briefly, immunoprecipitation reactions were performed with the above-indicated antibodies, each on approximately 500,000 cells, and incubated overnight at 4 °C. The immune complex was collected with protein A/G agarose or magnetic beads and washed sequentially in the low salt wash buffer (20 mM Tris pH8, 150 mM NaCl, 0.1% SDS, 1% Triton X-100, 2 mM EDTA), the high salt wash buffer (20 mM Tris pH8, 500 mM NaCl, 0.1% SDS, 1% Triton X-100, 2 mM EDTA), the LiCl wash buffer (10 mM Tris pH8, 250 mM LiCl, 1% NP-40, 1% Sodium Deoxycholate, 1 mM EDTA) and TE. Chromatin was eluted with elution buffer (1% SDS, 0.1 M NaHCO3), and then reverse cross-linked with 0.2 M NaCl at 65 °C for 4 h. DNA fragments were purified using Agencourt AMPure XP beads (Beckman Coulter, Brea, CA). Barcoded immunoprecipitated DNA and input DNA were prepared using the NEBNext ChIP-seq Library Prep Master Mix Set for Illumina (#E6240, New England Biolabs, Ipswich, MA) and TruSeq Adapters (Illumina) according to the manufacturer’s protocol on a SX-8G IP-STAR Compact Automated System (Diagenode). Phusion High-Fidelity DNA Polymerase (New England Biolabs) and TruSeq PCR Primers (Illumina, San Diego, CA) were used to amplify the libraries, which were then purified to remove adapter dimers using AMPure XP beads and multiplexed on the HiSeq 2000 (Illumina, San Diego, CA). Previously published CLL and normal B cells ChIP-seq datasets were downloaded from the Blueprint DCC portal (Blueprint; http://dcc.blueprint-epigenome.eu/#/home/).

ChIP-seq data were processed according to the ENCODE Histone ChIP-seq Data Standards and Processing Pipeline (https://www.encodeproject.org/chip-seq/histone/). Raw reads were mapped to the human genome GRCh37 assembly using Burrows-Wheeler Aligner^45^ (BWA v0.7.17). Duplicate reads were removed using Picard (https://broadinstitute.github.io/picard/) and bigwig files were created for visualization. Peaks were identified with Macs2^46^ (v2.0.10) with a *q*-value threshold of 0.01. De novo motif enrichment analyses were performed using Homer^47^ against JASPAR CORE database (-cpg parameter was used for CpG% normalization). Peaks overlapping with Satellite repeat regions and Encode Blacklist were discarded. All ChIP-seq reads were normalized and displayed as read counts per million mapped reads. Super-enhancers in H3K27ac peaks were defined as in^14,48^. First, for each sample, H3K27ac peaks without any overlap with known gene promoters (TSS ± 2.5 kb) were identified. Then, H3K27ac peaks within 12.5 kb of each other were concatenated and these regions were ranked by their total normalized H3K27ac signal. H3K27ac intensity was plotted against the corresponding concatenated enhancers rank. The cut-point between super-enhancers and enhancers was defined on the enrichment profile as the tangent with slope equal to 1. Enhancers on the right of the inflection point were defined as super-enhancers (see Supplementary Fig. 1c). We note that the number of super-enhancers identified in our CLL cohort (range [279–964]; median of 474 across samples) is in line with a recent study that investigated enhancer architecture in a distinct CLL cohort^48^. To identify variable super-enhancer domains enriched in either CLL or normal B cells, we defined the union of all super-enhancers discovered across the CLL and normal B cell cohorts. Differentially regulated super-enhancers in CLL compared with normal B cells (*n* = 297; see Fig. 1a–c and Supplementary Fig. 1d) were identified with DESeq2^49^ as those with absolute log_2_(H3K27ac fold-change) >2 and Benjamini-Hochberg adjusted *P*-value < 0.01. We note that few differences in the super-enhancer landscape were observed between the two major known CLL subtypes, even with less stringent fold-change and *P*-value thresholds (absolute log_2_[H3K27ac fold-change] >1 and Benjamini-Hochberg adjusted *P*-value < 0.05; see Supplementary Fig. 1f, g), consistent with prior studies showing more subtle chromatin differences between CLL subtypes^19,48^. For visualization purposes, count data matrices were transformed using variance stabilizing transformation (VST).

### RNA-seq

RNA was extracted using Qiagen (Hilden, Germany) RNeasy columns according to the manufacturer’s instructions. Subsequently, 500 ng of total RNA was used for polyA selection and TruSeq library preparation according to the instructions provided by Illumina (TruSeq RNA Sample Prep Kit v.2), with 8 cycles of PCR. Samples were barcoded and run on a HiSeq 4000 in a 125 bp paired-end mode, using the TruSeq SBS Kit v.3 (Illumina, San Diego, CA). An average of 75 million paired reads was generated per sample. Raw reads were mapped to the human genome GRCh37 using STAR (v2.5.2a) aligner^50^. We used several QC metrics for the RNA-seq library, including intron–exon ratio, intragenic reads fraction, and GC bias. We quantified exon and gene expression using Salmon^51^ against the *Homo sapiens* transcriptome GRCh37.

### Reduced representation bisulfite sequencing (RRBS)

Genomic DNA from CLL samples and normal B cell samples were used to produce RRBS libraries. These were generated by digesting genomic DNA with MspI to enrich for CpG-rich fragments, and then were ligated to barcoded TruSeq adapters (Illumina, San Diego, CA) to allow immediate subsequent pooling. This was followed by bisulfite conversion and PCR. Libraries were sequenced and 29mers were aligned to the hg19 genome using MAQ version 0.6.6^52^. Reads were further filtered if: (i) the read did not align to an autosome, (ii) the read failed platform/vendor quality checks (SAMtools flag 0 × 200), and/or (iii) the read did not align to an MspI cut site. The methylation state of each CpG was determined by comparing bisulfite-treated reads aligning to that CpG with the genomic reference sequence. The methylation level was computed by dividing the number of observed methylated cytosines (which did not undergo bisulfite conversion) by the total number of reads aligned to that CpG. In addition, the number of CpG measurements on each read was noted.

### Targeted bisulfite sequencing capture assay

Hybrid-selected sequencing libraries were prepared combining Accel-NGS Methyl-Seq DNA Library Kit (Swift Biosciences) with the NimbleGen SeqCap Epi Enrichment System (Roche NimbleGen), enabling lower input DNA quantities while maintaining library complexity. Briefly, pre-capture libraries were constructed following the “bisulfite-conversion first” library construction protocol of the Accel-NGS Methyl-Seq DNA Library Kit (Swift Biosciences) with the following exceptions: (1) To minimize the off-target sequencing rate, we sheared the input DNA to ~200 bp fragments instead of shearing to ~400 bp fragments; (2) we doubled the PCR volume and used 8 PCR cycles for the pre-capture library amplification in 1× HiFi HotStart ReadyMix (Kapa Biosystems). SeqCap Epi hybridization reactions contained a total of 1 µg of a pool of 2–4 PCR-amplified pre-capture libraries, a total of 1 nmol of 2–4 index-specific blocking oligonucleotides, and the custom SeqCap probe pool designed for the targets listed in Supplementary Data 4. Hybrid-selected sequencing libraries were sequenced on an Illumina HiSeq 2500 instrument in fast mode together with a 10% spike-in of a non-indexed PhiX174 library to generate a median of ~48 million indexed 100-base purity-filtered paired reads per sample. Raw reads were aligned to the human genome (hg19) using bsMap v2.9^53^ with the following parameters: bsmap -s 16 -v 0.1 -S 1 -n 1 -q 20 -r 0. Subsequently, we used Picard tools (http://picard.sourceforge.net) version 2.16.0 to further process and QC the aligned data files. Standard performance metrics for each library are available in Supplementary Data 5. We used MarkDuplicates with standard parameter settings to mark and remove likely PCR duplicates, CollectAlignmentMetrics to compute basic alignment statistics, and CalculateHsMetrics with Supplementary Data 4 to calculate all hybrid capture-related metrics, including the on-target rate (Supplementary Fig. 2a). To determine the methylation state of all CpGs captured and assess the bisulfite conversion rate, we used the mcall module in the MOABS^54^ software suite with standard parameter settings. Then, we converted the resulting CpG level files to bigBed files for visualization in IGV^55^, filtering out all CpGs that were covered with less than five reads. Analysis of targeted bisulfite sequencing capture assay data was conducted using the methylKit package^56^ and a 500-bp tiling of the target capture set. Briefly, we imported the CpG level methylation call files from mcall into R using the methylKit function “methRead” and then computed the weighted methylation mean for each 500-bp tile using the function “getData”, weighting the methylation level of each CpG with its coverage. We then merged the tile level methylation information across all samples and retained only those tiles covered with more than 10 reads in 70% or more of all samples. To compute differentially methylated tiles, we performed Fisher’s exact test on pooled CLL vs. normal B samples for each tile. Subsequently, we corrected the resulting *P*-values using Benjamini–Hochberg correction and defined regions with a *Q*-value ≤ 0.05 and an absolute methylation difference ≥ 0.3 as differentially methylated. Finally, we merged differentially methylated tiles into larger differentially methylated regions (DMRs) if they were less than 400 bp apart^22^.

BEDTools v2.25.0^57^ was used to calculate overlaps between differentially methylated regions with the different genomic features investigated, requiring a 50% minimal overlap fraction. Promoters were defined as 1 kb upstream and 1 kb downstream of hg19 RefGene gene transcription start sites (TSSs), unless stated otherwise. The set of CpG Islands (CGIs) were defined using biologically-verified CGIs^58^. ChIP-seq peak sets were defined as above-described. For the pathway analysis in Supplementary Fig. 2g, we used GREAT version 3.0.0^59^ to identify associated biological themes, using default association rule (i.e., basal plus extension: 5000 bp upstream, 1000 bp downstream; Hypergeometric test BH-FDR < 0.05).

### Whole-exome DNA sequencing (WES)

Genomic DNA from two *IGHV* mutated and two *IGHV* unmutated CLL patient samples were used to produce whole-exome libraries. Details of whole-exome library construction and analysis have been detailed elsewhere^3^. Briefly, output from Illumina software (Illumina, San Diego, CA) was processed by the Picard data processing pipeline to yield BAM files containing aligned reads with well-calibrated quality scores. We used the ABSOLUTE algorithm^60^ to calculate tumor purity—the ratio of tumor cells to total cells in the sample—and obtained a very high degree of purity (median of 0.98; range [0.9–1]), consistent with a negligible contamination of non-malignant cells in our CLL samples.

### Multiplexed single-cell RRBS (MscRRBS) library construction

Single cell experiments were performed by sorting DAPI negative cells in 96-well plates in 3 μL of 0.1× CutSmart buffer (New England Biolabs) per well using a BD Influx sorter (Becton Dickinson, Franklin Lakes, NJ). Nucleated CLL cells were gated and index-sorted as CD19^+^CD5^+^ cells, which in CLL patients are overwhelmingly malignant^61^ (≥95%). Plates were stored at −80 °C until further processing. The day of the experiment, cells were lysed for 2 h at 50 °C in 1× CutSmart buffer supplemented with Proteinase K (0.2U, NEB) and Triton X-100 (0.3%, Sigma Aldrich) for a final volume of 5 μL. Proteinase K was heat-inactivated for 30 min at 75 °C. DNA was incubated with 10 units of the restriction enzyme Msp1 (Fermentas) in 6.5 μL final volume reaction during 90 min at 37 °C. Heat-inactivation was performed for 10 min at 70 °C. Digested DNA was filled-in and A-tailed at the 3′ sticky ends in 8.5 μL final volume of 1× CutSmart with 2.5 units of Klenow fragment (Exo-, Fermentas). Reaction was supplemented with 1 mM dATP and 0.1 mM dCTP and 0.1 mM dGTP (NEB) and performed as follows in a thermocycler: 30 °C for 25 min, 37 °C for 25 min and heat-inactivation at 70 °C for 10 min. Custom barcoded methylated adapters (0.1 μM) were then ligated overnight at 16 °C with the dA-tailed DNA fragments in the presence of 800 units of T4 DNA ligase (NEB) and 1 mM ATP (Roche) in a final volume of 11.5 μL of 1× CutSmart buffer. T4 DNA ligase heat-inactivation was performed at 70 °C for 15 min the next day. Genomic DNA from 24 individual cells were pooled together according to their barcodes, giving, for a 96-well plate, 4 pools of 24 cells. Pooled genomic DNA was cleaned-up and concentrated using 1.8× SPRI beads (Agencourt AMPure XP—Beckman Coulter). Each pool was then sodium bisulfite converted (Fast Epitect Bisulfite, Qiagen) following manufacture recommendations. To ensure full bisulfite conversion, two cycles of conversion were performed. The double-stranded DNA was first denatured 10 min at 98 °C and then incubated for 20 min at 60 °C. Hundred nanogram of dephosphorylated and sheared bacterial DNA was added as carrier to every pool prior to conversion. Converted DNA was then amplified using primers containing Illumina i7 and i5 index. Following Illumina pooling guidelines, a different i7 index was used for every 24-cell pool, allowing multiplexing of 96 cells for sequencing on one Illumina HiSeq lane. Library enrichment was done using KAPA HiFi Uracil + master mix (Kapa Biosystems) and the following PCR condition was used: 98 °C for 45 s; 6 cycles of: 98 °C for 20 s, 58 °C for 30 s, 72 °C for 1 min; followed by 12 cycles of: 98 °C for 20 s, 65 °C for 30 s, 72 °C for 1 min. PCR was terminated by an incubation at 72 °C for 5 min. Enriched libraries were cleaned-up and concentrated using 1.3X SPRI beads. DNA fragments between 200 bp and 1 kb were size-selected and recovered after resolving on a 3% NuSieve 3:1 agarose gel. Libraries molarity concentration calculation was obtained by measuring concentration of double stranded DNA (Qubit) and quantifying the average library size (bp) using an Agilent Bioanalyzer. Every 24-cells pool was mixed with the others pool in an equimolar ratio. All cells from a 96-well plate were sequenced as paired-end on HiSeq 2500 with 10% PhiX spike-in. Negative controls (empty wells with no cell) were used to control for non-specific amplification of the libraries.

### MscRRBS read alignment

Each pool of 96 cells was first demultiplexed by Illumina i7 barcodes (Supplementary Data 11), resulting in four pools of 24 cells. Each pool of 24 cells was further demultiplexed by unique cell barcodes (Supplementary Data 12). Reads were assigned to a given cell if they matched 80% of the template adapters. Adapters and adapter reverse complements (6 bp) were trimmed from the raw sequence reads. After adapter removal, reads were trimmed from their 3′ end for read quality by applying a 4 bp sliding window and removing bases until the mean base quality of the window had a Phred quality score greater than 15. Read pairs with a read shorter than 36 bp after trimming were discarded. We aligned trimmed reads in single-end mode to the hg19 human genome assembly using Bismark^62^ (v.0.14.5; parameters: -multicore 4 -X 1000 --un –ambiguous) running on bowtie2-2.2.8 aligner^63^. Bismark methylation extractor (--bedgraph --comprehensive) was used to determine the methylation state of each individual CpG. For downstream analyses, a site was considered methylated or unmethylated only if there was 90% agreement of the methylation state for all reads mapped to the site.

### Joint MscRRBS and single-cell RNA-seq library construction

Single cells were sorted by flow cytometry into 2.5 μL of RLT Plus buffer (Qiagen) supplemented with 1 U/μL of RNase Inhibitor (Lucigen). Sorted cells were immediately stored at −80 °C. Genomic DNA (gDNA) and mRNA have been separated manually. A modified oligo-dT primer (5′-biotin-triethyleneglycol-AAGCAGTGGTATCAACGCAGAGTACT30VN-3′, where V is either A, C or G, and N is any base; IDT) was conjugated to streptavidin-coupled magnetic beads (Dynabeads, Life Technologies) according to the manufacturer’s instructions. To capture polyadenylated mRNA, we added the conjugated beads (10 μL) directly to the cell lysate and incubated them for 20 min at room temperature with mixing to prevent the beads from settling. The mRNA was then collected to the side of the well using a magnet, and the supernatant, containing the gDNA, was transferred to a fresh plate. Single-cell complementary DNA was amplified from the tubes containing the captured mRNA according to the Smart-seq2 protocol^64^. After amplification and purification using 0.8× SPRI beads, 0.5 ng cDNA was used for Nextera Tagmentation and library construction. Library quality and quantity was respectively assessed using Agilent Bioanalyzer 2100 and Qubit, respectively. Genomic DNA present in the pooled supernatant and wash buffer from the mRNA isolation step was precipitated on 0.8× SPRI beads and eluted directly into the reaction mixtures for Msp1 (Fermentas) enzymatic reaction (10 μL final reaction). MscRRBS protocol was then performed on the digested gDNA after the restriction enzyme digestion step.

### Single-cell RNA-seq gene expression quantification

The sequenced read fragments were mapped against the hg19 human genome assembly using the 2pass default mode of STAR^50^ (version 2.5.2a) with the annotation of GENCODE^65^ (version 19). The number of read counts overlapping with annotated genes were quantified applying the “GeneCounts” option in the STAR alignment. We filtered out poor quality cells when the detected number of genes was below 500 or the fraction of mitochondrial gene counts was higher than 20%. To compare the cells in terms of their transcriptional differences, we normalized the read counts by scaling for the total number of counts per cell. To assess potential confounding effect due to cell cycle phase, we classified CLL cells into cell cycle phases using AUCell method implemented in the SCENIC analytical toolkit^66^. Briefly, AUCell uses the area under the curve (AUC) to identify cells with active gene sets, by calculating the proportion of genes in each input gene set that is enriched within the expressed genes for each cell. Each cell is assigned an AUC score for each gene set. Cells expressing many genes from the gene set will have higher AUC score than cells expressing fewer. Last, the highest AUC threshold is used to consider a gene set “active” in a given cell. The Molecular Signature Database^67^ (MSigDB; http://www.broad.mit.edu/gsea/) C2 curated gene sets “BIOCARTA_G2_PATHWAY” and “BIOCARTA_G1_PATHWAY” were used as input gene sets for this analysis. We observed that the vast majority of cells are classified as being non-cycling cells (*n* = 240; 99.6%), with a negligible number of cells being in either G2/M (*n* = 1; 0.4%; AUC > 0.27) or G1 phase (*n* = 0; 0%; AUC > 0.18), consistent with the majority of CLL cells being in a resting non-cycling state^68^.

### Single-cell DNAme-gene expression MI analysis

To begin, cells with fewer than 500 detected genes or a proportion of mitochondrial or ribosomal reads above 20% were removed from the analysis for quality control. Constitutively highly expressed mitochondrial genes and genes encoding ribosomal proteins across all cells were then removed. Then, cells in the bottom 10th percentile of total read counts for a given sample were discarded, and each of the remaining cells was probabilistically downsampled to match the number of reads at this cutoff. Subsequently, genes with reads detected in less than five cells were removed from the analysis. At single-cell resolution, a gene’s promoter methylation rate was represented by the proportion of methylated CpGs in the region 2500 base pairs upstream and downstream of the transcription start site. Genes with less than 10 CpG observations in the promoter region for a given cell were removed. We then computed the mutual information between promoter methylation rate and gene expression for each cell using a threshold of zero, implying that any detected methylation or expression for a given gene was treated as having a value of 1 for that cell, and 0 otherwise. For a gene to be included in the final analysis, it was required to have at least 10 cells with sufficient CpG data for a methylation call (10 CpG observations) as well as greater than 10% non-zero expression across all cells to mitigate the impact of dropout. The approach was validated by a non-parametric premutation test, in which we randomly permuted the cell methylation values for each gene while holding the corresponding expression vector constant (such that RNA and DNAme are no longer linked at the single-cell level) and computed an unmatched version of the mutual information. This was repeated as many times as cells were available for a given gene, and the final unmatched mutual information value provided corresponds to the median of the result for each of these permutations. We note that the analysis for Fig. 3c was performed with downsampling to create a balanced dataset by matching the number of genes between CLL and normal B cells (*n* = 759 genes).

### Gene set enrichment analysis

Gene set enrichment analysis was performed using GSEA software, and Molecular Signature Database^67^ (MSigDB; http://www.broad.mit.edu/gsea/). Specifically, we used the C2 curated gene sets and Benjamini-Hochberg FDR adjusted *P*-value cut-off of 0.05.

### Chromatin hidden Markov model (HMM)

Chromatin states across the genome were defined using EpicSeg^69^, which is based on a multivariate HMM, using H3K4me3, H3K27ac, H3K27me3, whole cell extract, RNA-seq and DNAme (based on bulk RRBS) datasets as input. ChIP-seq reads were shifted in the 5′–3′ direction by 100 bp. Reads counts were computed in 200 bp non-overlapping bins. Normalized raw counts were then modeled with an HMM assuming that the hidden state vector followed a negative binomial distribution. We trained several HMM models in parallel mode with the number of states ranging from 5 states to 25 states and chose a 12-state model as the best model that captures all the key interactions between the epigenetic marks and cover all possible genomic elements (promoter, enhancer, gene body) that we expected to resolve given the selection of datasets we used (H3K4me3, H3K27ac, H3K27me3, RNA-seq, and DNAme). Genomic regions were then annotated with the state with the maximum posterior probability in the 200 bp bin. State enrichment in different genomic features was calculated dividing the percentage of nucleotides occupied by a state in a particular genomic feature by the percentage of nucleotides that this genomic feature represents in the entire genome.

### Chromatin Dirichlet process Gaussian mixture model

Infinite mixture model with the Dirichlet process was used to model the normalized signal count matrix and to derive a segmentation of the chromatin tracks. The scikit-learn Python library (sklearn.mixture.BayesianGaussianMixture v0.19) was used to generate an independent model for each sample. Cross-validation for each sample was performed training on a random 1/10 of the genome, applying the cross-validation model to the sample and repeating this procedure 100 times. Subsequently, a leave-one-out procedure was implemented to assess the contribution of each chromatin and transcriptome track independently. Unsupervised hierarchical clustering of state emission was performed to identify unique states.

### Single-cell entropy analysis

To test for significance of association of chromatin state status with expression heterogeneity in Figs. 3f and 4g, single cell RNA-seq read counts observed in each cell were normalized by the effective library size and transcript length, and the fraction of positive cells (fpc) was calculated per gene (a cell is defined as positive if > 0 reads aligned to the gene). Subsequently, Shannon’s information entropy (ent) was calculated for each gene as followed:

1 $${\mathrm{ent}}=\left[-1 \times \left( {\mathrm{fpc}}\, \times {\mathrm{log}}_{2}({\mathrm{fpc}}) + (1- {\mathrm{fpc}})\times {\mathrm{log}}_{2}\left(1- {\mathrm{fpc}}\right)\right)\right]$$

The association with chromatin state status was tested using a generalized additive model (implemented by gam R package). The following type of model was tested:

$${\mathrm{ent}} \sim {\mathrm{s}}\left({\mathrm{population}}\, {\mathrm{average}}\, {\mathrm{expression}}\right) + {\mathrm{chromatin}}\, {\mathrm{state}}\, {\mathrm{status}}$$

where s() indicates local regression. The population average expression values were entered into the model on a log_10_ scale.

### Statistical methods

Statistical analysis was performed with Python 2.7.13 and R version 3.4.2. Categorical variables were compared using the Fisher’s Exact test. Continuous variables were compared using the Mann–Whitney *U*-test, Welch’s *t*-test, paired *t*-test, non-parametric permutation test or Kolmogorov–Smirnov test as appropriate. *P*-values were adjusted for multiple comparisons by Benjamini-Hochberg FDR procedure, as appropriate. All *P*-values are two-sided and considered significant at the 0.05 level unless otherwise noted.

### Reporting summary

Further information on experimental design is available in the Nature Research Reporting Summary linked to this article.

## Supplementary information

Supplementary Information

Description of Additional Supplementary Files

Supplementary Data 1

Supplementary Data 2

Supplementary Data 3

Supplementary Data 4

Supplementary Data 5

Supplementary Data 6

Supplementary Data 7

Supplementary Data 8

Supplementary Data 9

Supplementary Data 10

Supplementary Data 11

Supplementary Data 12

Reporting Summary

## Data Availability

ChIP-seq, RNA-seq, and DNAme datasets have been deposited to the NCBI Gene Expression Omnibus^70^ (GEO) under accession number GSE119103. MscRRBS and single-cell Smart-seq2 datasets have been deposited to the NCBI GEO under accession number GSE109085. The dbGaP accession number for the whole-exome sequencing data reported in this paper is phs000435.v2.p1. H3K27me3 ChIP-seq data for primary human tonsillar naive B cells and tonsillar germinal center B cells were downloaded from NCBI GEO under accession number GSE45982^50^. Previously published CLL and normal B cell ChIP-seq and RNA-seq datasets were downloaded from the Blueprint DCC portal under accession number EGAC00001000135.
