# Supplementary material for: Corrupted coordination of epigenetic modifications leads to diverging chromatin states and transcriptional heterogeneity in CLL
Source: Nat Commun. 2019 Apr 23;10:1874. doi: 10.1038/s41467-019-09645-5 (PMC6478836; doi:10.1038/s41467-019-09645-5)
Supplement: Supplementary file 1 — Supplementary Information [file 41467_2019_9645_MOESM1_ESM.pdf]

Supplementary Information

Corrupted coordination of epigenetic modifications leads to diverging chromatin states and transcriptional heterogeneity in CLL

Pastore A, Gaiti F, *et al*.

a.

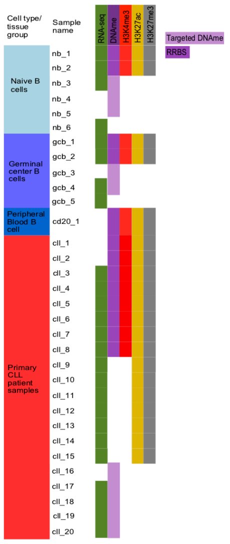

b.

Samples ID	Cell type	Surface markers	Source	*IGHV* status	FISH cytogenetics
nb_1	Naive B cell	CD19⁺CD23⁺CD27⁻IgD⁺	Tonsil	NA	NA
nb_2	Naive B cell	CD19⁺CD23⁺CD27⁻IgD⁺	Tonsil	NA	NA
nb_3	Naive B cell	CD19⁺CD23⁺CD27⁻IgD⁺	PB	NA	NA
nb_4	Naive B cell	CD19⁺CD23⁺CD27⁻IgD⁺	PB	NA	NA
nb_5	Naive B cell	CD19⁺CD23⁺CD27⁻IgD⁺	PB	NA	NA
nb_6	Naive B cell	CD19⁺CD23⁺CD27⁻IgD⁺	PB	NA	NA
gcb_1	Germinal center B cell	CD19⁺CD23⁺CD27⁺IgD⁻	Tonsil	NA	NA
gcb_2	Germinal center B cell	CD19⁺CD23⁺CD27⁺IgD⁻	Tonsil	NA	NA
gcb_3	Memory B cell	CD19⁺CD23⁺CD27⁺IgD⁻	PB	NA	NA
gcb_4	Memory B cell	CD19⁺CD23⁺CD27⁺IgD⁻	PB	NA	NA
gcb_5	Memory B cell	CD19⁺CD23⁺CD27⁺IgD⁻	PB	NA	NA
cd20_1	B cell	CD19⁺CD20⁺CD38⁺⁺	Tonsil	NA	NA
cll_1	CLL	NA	PB	mutated	del(13q)
cll_2	CLL	NA	PB	mutated	del(6q21), del(13q)
cll_3	CLL	NA	PB	mutated	del(13q)
cll_4	CLL	NA	PB	mutated	normal (46,XX)
cll_5	CLL	NA	PB	mutated	normal (46,XX)
cll_6	CLL	NA	PB	unmutated	del(11q), del(13q), amp(2p)
cll_7	CLL	NA	PB	unmutated	del(13q)
cll_8	CLL	NA	PB	mutated	del(13q)
cll_9	CLL	NA	PB	mutated	del(13q)
cll_10	CLL	NA	PB	mutated	del(13q)
cll_11	CLL	NA	PB	mutated	normal (46,XY)
cll_12	CLL	NA	PB	unmutated	del(13q)
cll_13	CLL	NA	PB	mutated	del(11q), del(13q)
cll_14	CLL	NA	PB	mutated	normal (46,XY)
cll_15	CLL	NA	PB	unmutated	NA
cll_16	CLL	NA	PB	mutated	del(13q)
cll_17	CLL	NA	PB	mutated	del(13q)
cll_18	CLL	NA	PB	mutated	normal (46,XX)
cll_19	CLL	NA	PB	unmutated	Tri12
cll_20	CLL	NA	PB	unmutated	del(11q)

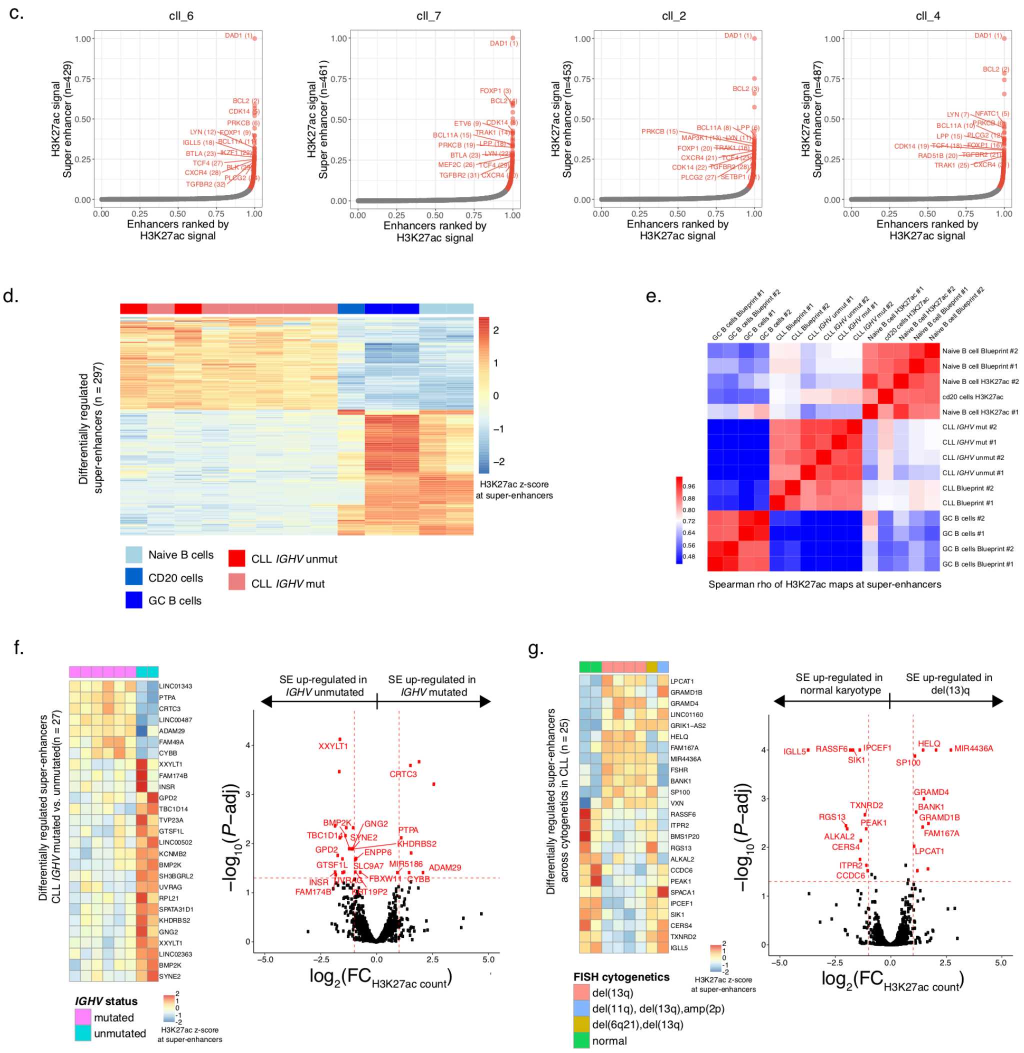

Supplementary Figure 1. H3K27ac analysis of CLLs and normal B cell controls at super-enhancers.

(a) Overview of samples used in this study across our cohort. See Supplementary Data 1 for the list of additional CLL and normal B cell samples used in this study from the Blueprint Initiative. **(b)** Summary table of healthy B cell and CLL patient samples used in this study across our cohort, including cell type, surface markers, source, *IGHV* mutational status, FISH cytogenetics and CLL driver mutations. PB: Peripheral Blood. **(c)** Enhancer profiles for four representative CLL samples, using H3K27ac ChIP-seq signal. Super-enhancers (SE) are highlighted in red with ranks of selected SE-associated genes. See Methods for SE identification criteria. **(d)** Extended heatmap of H3K27ac profiles shown in Fig. 1a for 297 differentially regulated super-enhancers (absolute log_2[H3K27ac fold-change] > 2 and Wald test BH-FDR < 0.01) between CLL and normal B cells. Red indicates high H3K27ac level, blue low H3K27ac level. **(e)** Spearman's rho correlation coefficients of H3K27ac enrichment signal at super-enhancers across our cohort and Blueprint initiative samples. Red indicates high Spearman's correlation, blue indicates low Spearman's correlation. **(f)** *Left*: Heatmap of H3K27ac profiles for 27 differentially regulated super-enhancers (absolute log_2[H3K27ac fold-change] > 1 and Wald test BH-FDR < 0.05) between CLL *IGHV* mutated and unmutated samples. Red indicates high H3K27ac level, blue low H3K27ac level. *Right*: volcano plot of differential H3K27ac signal at super-enhancers between CLL *IGHV* mutated and unmutated samples. **(g)** Same as panel **(f)** for 25 differentially regulated super-enhancers (absolute log2[H3K27ac fold-change] > 1 and Wald test BH-FDR < 0.05) between CLL patient samples with common somatic copy number abnormalities [del(13q), del(11q), del(17p), del(6q), amp(2p)] and normal karyotype. Cytogenetics were evaluated by FISH analysis (see Methods).

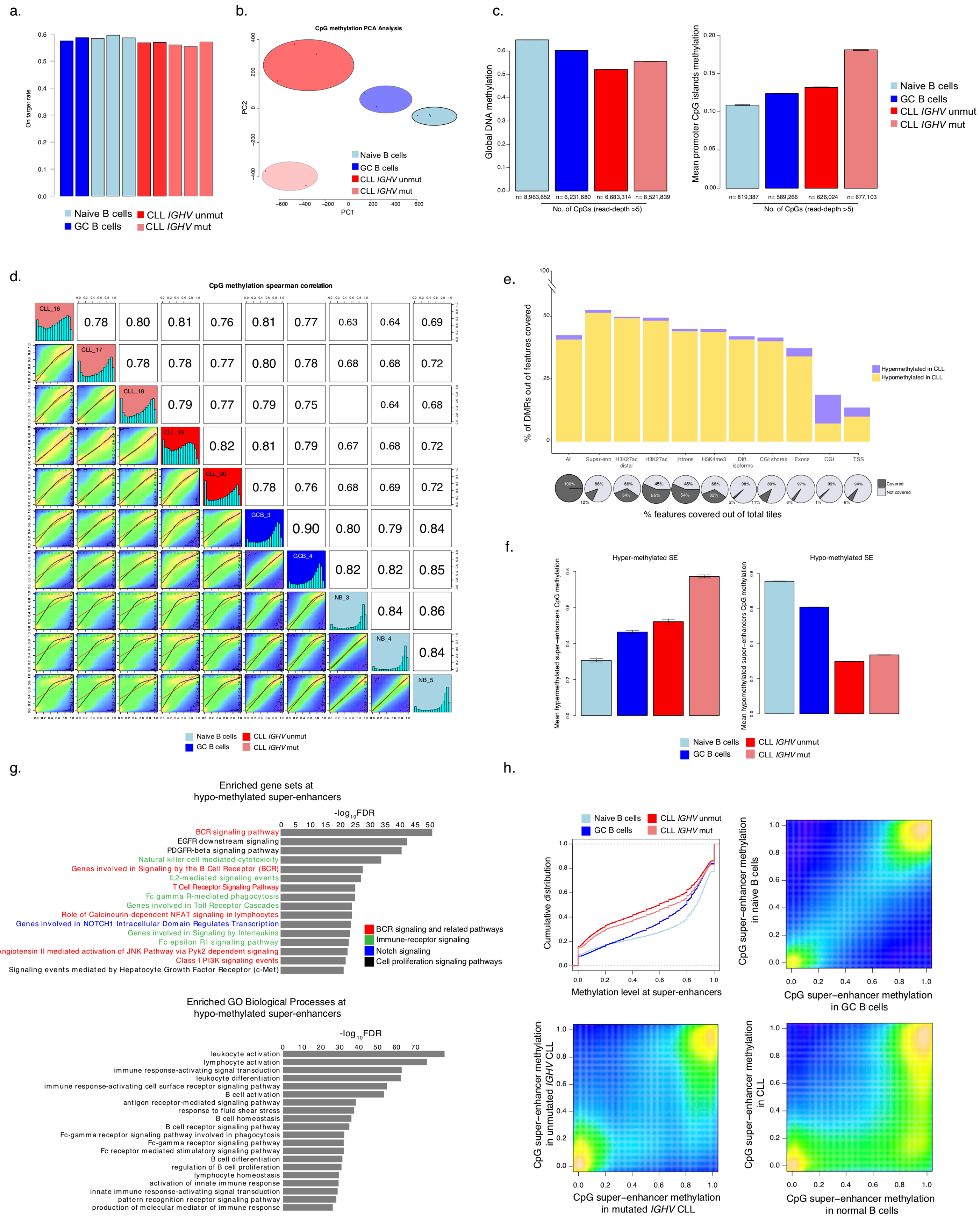

a.
On target rate
Naive B cells
GC B cells
CLL IGHV unmut
CLL IGHV mut
b.
CpG methylation PCA Analysis
PC2
PC1
Naive B cells
GC B cells
CLL IGHV unmut
CLL IGHV mut
c.
Global DNA methylation
n= 8,963,652
n= 6,231,680
n= 6,683,314
n= 8,521,839
No. of CpGs (read-depth >5)
Mean promoter CpG islands methylation
n= 819,387
n= 589,266
n= 626,024
n= 677,103
No. of CpGs (read-depth >5)
Naive B cells
GC B cells
CLL IGHV unmut
CLL IGHV mut
d.
CpG methylation spearman correlation
CLL_16
CLL_17
CLL_18
CLL_19
CLL_20
GCB_3
GCB_4
NB_3
NB_4
NB_5
0.78 0.80 0.81 0.76 0.81 0.77 0.63 0.64 0.69
0.78 0.78 0.77 0.80 0.78 0.68 0.68 0.72
0.79 0.77 0.79 0.75 0.64 0.68
0.82 0.81 0.79 0.67 0.68 0.72
0.78 0.76 0.68 0.69 0.72
0.90 0.80 0.79 0.84
0.82 0.82 0.85
0.84 0.86
0.84
Naive B cells
GC B cells
CLL IGHV unmut
CLL IGHV mut
e.
% of DMRs out of features covered
All
Super-enh
H3K27ac distal
H3K27ac
Introns
H3K4me3
Diff. isoforms
CGI shores
Exons
CGI
TSS
Hypermethylated in CLL
Hypomethylated in CLL
Covered
Not covered
% features covered out of total tiles
f.
Hyper-methylated SE
Mean hypermethylated super-enhancers CpG methylation
Hypo-methylated SE
Mean hypomethylated super-enhancers CpG methylation
Naive B cells
GC B cells
CLL IGHV unmut
CLL IGHV mut
g.
Enriched gene sets at hypo-methylated super-enhancers
-log10FDR
BCR signaling pathway
EGFR downstream signaling
PDGFR-beta signaling pathway
Natural killer cell mediated cytotoxicity
Genes involved in Signaling by the B Cell Receptor (BCR)
IL2-mediated signaling events
T Cell Receptor Signaling Pathway
Fc gamma R-mediated phagocytosis
Genes involved in Toll Receptor Cascades
Role of Calcineurin-dependent NFAT signaling in lymphocytes
Genes involved in NOTCH1 Intracellular Domain Regulates Transcription
Genes involved in Signaling by Interleukins
Fc epsilon RI signaling pathway
Angiotensin II mediated activation of JNK Pathway via Pyk2 dependent signaling
Class I PI3K signaling events
Signaling events mediated by Hepatocyte Growth Factor Receptor (c-Met)
BCR signaling and related pathways
Immune-receptor signaling
Notch signaling
Cell proliferation signaling pathways
Enriched GO Biological Processes at hypo-methylated super-enhancers
-log10FDR
leukocyte activation
lymphocyte activation
immune response-activating signal transduction
leukocyte differentiation
immune response-activating cell surface receptor signaling pathway
B cell activation
antigen receptor-mediated signaling pathway
response to fluid shear stress
B cell homeostasis
B cell receptor signaling pathway
Fc-gamma receptor signaling pathway involved in phagocytosis
Fc-gamma receptor signaling pathway
Fc receptor mediated stimulatory signaling pathway
B cell differentiation
regulation of B cell proliferation
lymphocyte homeostasis
activation of innate immune response
innate immune response-activating signal transduction
pattern recognition receptor signaling pathway
production of molecular mediator of immune response
h.
Naive B cells
GC B cells
CLL IGHV unmut
CLL IGHV mut
Cumulative distribution
Methylation level at super-enhancers
CpG super-enhancer methylation in naive B cells
CpG super-enhancer methylation in GC B cells
CpG super-enhancer methylation in unmutated IGHV CLL
CpG super-enhancer methylation in mutated IGHV CLL
CpG super-enhancer methylation in CLL
CpG super-enhancer methylation in normal B cells

Supplementary Figure 2. Targeted bulk DNAme sequencing capture assay of CLLs and normal B cell controls.

(**a**) On-target rate of the hybrid capture DNAme probes across all CLL (*IGHV* unmutated, n = 2; *IGHV* mutated, n = 3) and normal B (peripheral blood naïve B cells, n = 3; peripheral blood memory B cells, n = 2) samples. (**b**) Unsupervised principal component analysis (PCA) of targeted DNAme sequencing capture assay data for CLL and normal B samples. Same samples as in panel **(a)** were used. PCA was computed by filtering rows with a standard deviation lower than the 50th percentile of standard deviation distribution. (**c**) Global (*left*) and promoter CpG islands (*right*) mean CpG methylation in pooled CLL compared with pooled normal B samples, measured with targeted bisulfite sequencing capture assay. Same samples as in panel **(a)** were used. (**d**) Scatterplots of pairwise Spearman correlation between CLL and normal B samples. Same samples as in panel **(a)** were used. The red line in the plot is from linear regression fit and the green line is from LOWESS polynomial regression fit. (**e**) Percentage of differentially methylated regions (DMRs) covered by targeted bisulfite sequencing capture assay that overlap with distinct genomic features. (**f**) CLL CpG methylation compared with normal B cells at hyper-methylated super-enhancers in CLL (n = 122, *left*) and hypo-methylated super-enhancers in CLL (n = 2009, *right*). (**g**) *Top*: Gene set enrichment for CLL hypomethylated super-enhancers. *Bottom*: Gene ontology enrichment for CLL hypomethylated super-enhancers. See Supplementary Data 9 for complete list of significantly enriched gene sets. (**h**) Cumulative distribution of CpG methylation values in CLL and normal B cells at super-enhancers (n = 2,869) (*top left*). Scatter plots comparing methylation pattern consistency at super-enhancers of two normal B cell samples (*top right*), and two CLL samples (*bottom left*). A comparison between CpG methylation levels at super-enhancers in CLL (no. of CpGs used = 468,303) and normal B cells (no. of CpGs used = 502,607) is also shown (*bottom right*). Throughout the figure, error bars represent 95% confidence interval.

a.

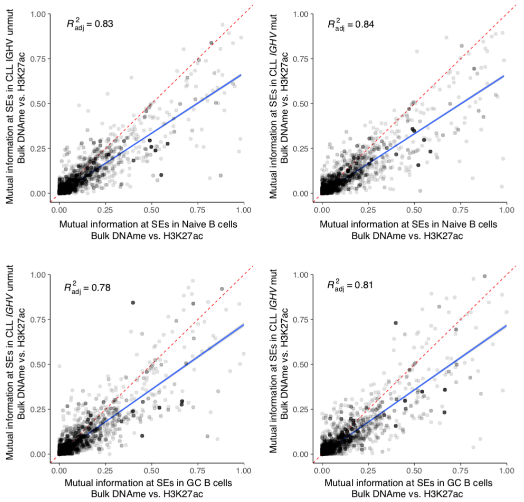

b.

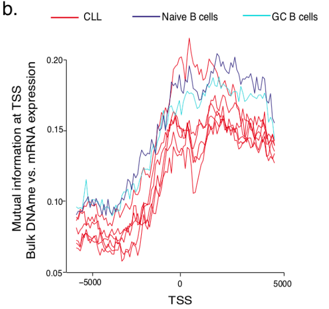

c.

Samples ID	Cell type	Surface markers	*IGHV* status	No.of single cells
scBcell	Normal B cell	CD19⁺	NA	96
scCLL_21	CLL	CD19⁺CD5⁺	unmutated	96
scCLL_22	CLL	CD19⁺CD5⁺	mutated	192

d.

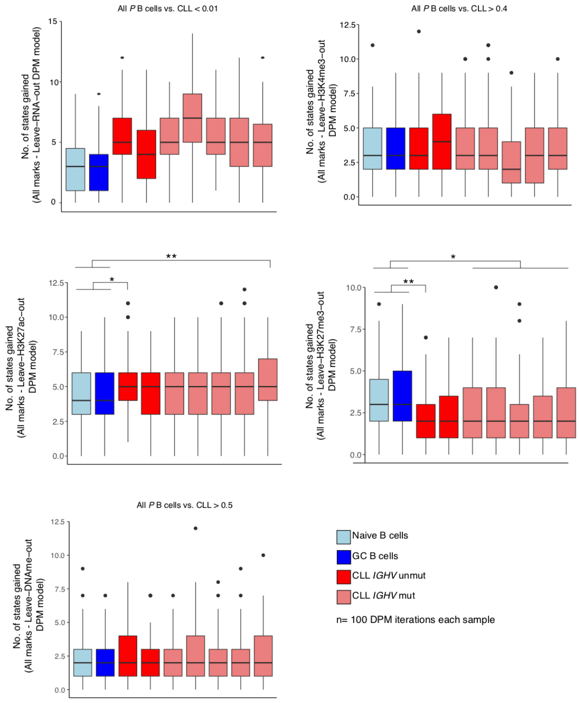

e.

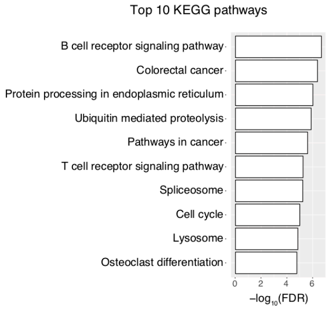

f.

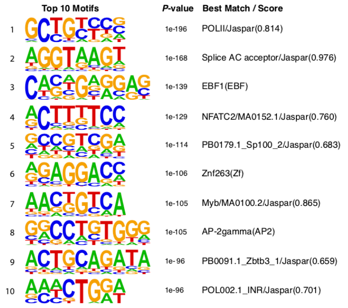

g.

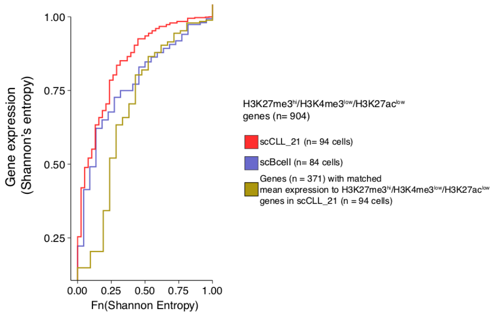

Supplementary Figure 3. Normal B cells exhibit higher coordinated transcriptional regulation compared with CLL.

(a) Regression analysis of mutual information (MI) at super-enhancers (n = 2,869) between bulk DNAme based on targeted bisulfite sequencing capture assay and H3K27ac for CLL (y-axes) and normal B (x-axes) samples. **(b)** MI at transcription start sites of genes between DNAme (based on bulk bisulfite sequencing) and gene expression in CLL and normal B samples. **(c)** Summary table of healthy donors and CLL patient single cell samples used in this study, including the cell type, surface markers, *IGHV* mutational status and number of single cells sequenced. **(d)** From *left* to *right:* number of states gained when adding bulk RNA-seq data to the epigenomic mapping (H3K4me3, H3K27ac, H3K27me3, bulk DNAme based on bisulfite sequencing) in the DPM analysis for CLL and normal B samples; number of states gained when adding H3K4me3 data to the epigenomic mapping (bulk RNA-seq data, H3K27ac, H3K27me3, bulk DNAme); number of states gained when adding H3K27ac data to the epigenomic mapping (bulk RNA-seq data, H3K4me3, H3K27me3, bulk DNAme); number of states gained when adding H3K27me3 data to the epigenomic mapping (bulk RNA-seq data, H3K27ac, H3K4me3, bulk DNAme); number of states gained when adding DNAme data to the epigenomic mapping (bulk RNA-seq data, H3K27ac, H3K27me3, H3K4me3). Boxplots represent median and bottom and upper quartile. Lower and upper whiskers correspond to 1.5*IQR. * indicates two-sided Mann-Whitney U test *P*-value < 0.05. ** indicates *P*-value < 0.01. 100 DMP iterations were performed for each sample. **(e)** KEGG pathways enriched at genes marked by H3K27me3^{hi}/H3K4me3^{low}/H3K27ac^{low} from Fig. 3e. Shown are the top 10 KEGG pathway categories (hypergeometric test BH-FDR < 0.05). (**f**) Position weight matrices of the top 10 motifs over-represented in CLL in regions marked by H3K27me3^{hi}/H3K4me3^{low}/H3K27ac^{low} from Fig. 3e. Motif enrichment hypergeometric test *P*-value and the best match to the reference motif in the JASPAR core database are also shown. (**g**) Cumulative distribution of single-cell gene expression Shannon's information entropy for H3K27me3^{hi}/H3K4me3^{low}/H3K27ac^{low}-marked genes in CLL (red; n = 94) and normal B (blue; n = 84) cells, along with genes (n = 371) with matched mean expression to H3K27me3^{hi}/H3K4me3^{low}/H3K27ac^{low}-marked genes in CLL (yellow).

a.

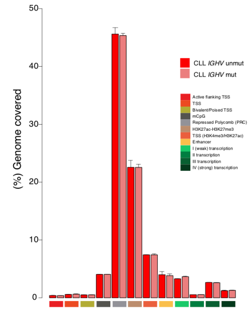

b.

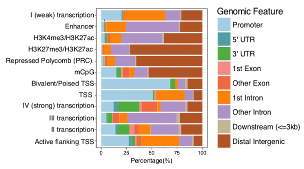

c.

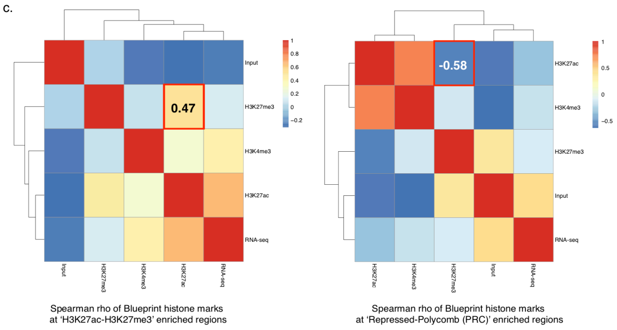

d.

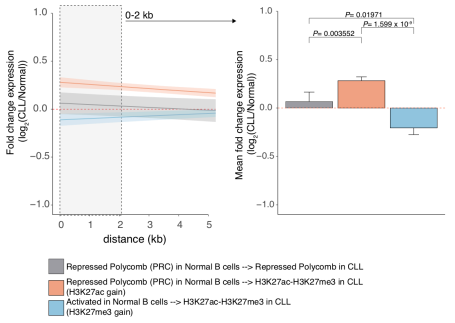

e.

Histone H3 modification transition	Gene sets (CGP) enriched in closest genes to genomic segments that gain H3K27ac from normal B cells to CLL	BH-FDR
H3K27ac gain	ES with H3K27me3 (Benporath)	2.48×10^{-45}
H3K27ac gain	Methylated in liver cancer DN (Acevedo)	2.20×10^{-38}
H3K27ac gain	Stem cell UP (Boquest)	4.06×10^{-38}
H3K27ac gain	Mammary stem cell UP (Lim)	2.26×10^{-33}
H3K27ac gain	Adult tissue stem module (Wong)	5.68×10^{-30}
H3K27ac gain	Hematopoietic stem cell UP (Jaatinen)	7.18×10^{-28}
H3K27ac gain	Ewing sarcoma progenitor UP (Riggi)	9.79×10^{-25}
H3K27ac gain	UVC response via TP53 group A (Bruins)	3.05×10^{-23}
H3K27ac gain	Liver cancer with H3K27me3 UP (Acevedo)	2.97×10^{-21}
H3K27ac gain	Stem cell cultured vs. fresh UP (Boquest)	2.20×10^{-20}

f.

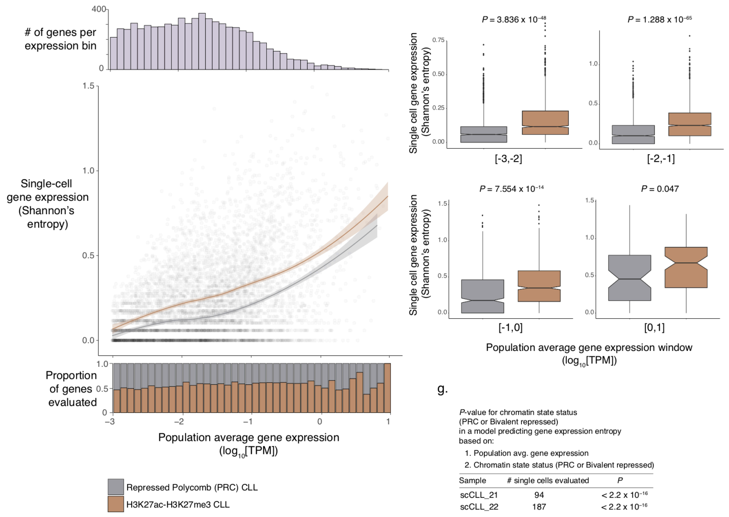

g.

P-value for chromatin state status
(PRC or Bivalent repressed)
in a model predicting gene expression entropy
based on:

1. Population avg. gene expression
2. Chromatin state status (PRC or Bivalent repressed)

Sample	# single cells evaluated	*P*
scCLL_21	94	$< 2.2 \times 10^{-16}$
scCLL_22	187	$< 2.2 \times 10^{-16}$

Supplementary Figure 4. Decreased coordination between different layers of the CLL epigenome and increased cell-to-cell transcriptional heterogeneity.

(**a**) Genomic coverage (%) of chromatin states from Fig. 4a in CLL *IGHV* mutated and *IGHV* unmutated, showing no significant difference between the two disease subtypes. (**b**) Percentage of genomic feature covered by each chromatin state from Fig. 4a. **(c)** Spearman's rho correlation coefficients of histone marks enrichment signal across Blueprint initiative samples at 'H3K27ac-H3K27me3' regions (*left*) and 'Repressed Polycomb (PRC)' regions (*right*) identified in our data. Red indicates high correlation, blue low correlation. (**d**) Fold-change gene expression [log_2(RPKM)] between CLL and normal B cells in relation to genomic distance (kb) from regions that gain H3K27ac in CLL (orange; n = 11,740 genes), regions that gain H3K27me3 in CLL (blue; n = 8,867 genes), and regions that did not exhibit chromatin state transition between normal B and CLL cells (grey, n = 833 genes). *P*-values are shown for two-sided Mann-Whitney U test. (**e**) Gene sets (CGP) enriched in closest genes (average distance of 496 bp) to genomic segments that gain H3K27ac from normal B cells to CLL (hypergeometric test BH-FDR < 0.05; see Supplementary Data 10 for the top 50 enrichments). **(f)** *Left*: Single-cell gene expression Shannon's information entropy (y-axis) in relation to the population average gene expression (x-axis, log_{10}[TPM]) in scCLL_21 single cells (n = 94). Colored lines – local regression curves for genes in a 'H3K27ac-H3K27me3' (brown) or 'Repressed Polycomb (PRC)' (grey) state. *Right*: Single-cell gene expression Shannon's information entropy for each of the two HMM chromatin states for genes with population average gene expression of [-3,-2], [-2,-1], [-1,0], [0,1] log_{10}[TPM] (to control for differences in this variable), respectively. Boxplot represents median and bottom and upper quartile. Lower and upper whiskers correspond to 1.5*IQR. *P*-values are shown for two-sided Mann-Whitney U test. **(g)** Generalized additive regression tests that model single-cell gene expression Shannon's information entropy based on population average gene expression and chromatin state status across the 2 CLL samples that underwent single-cell whole-transcriptome sequencing. Throughout the figure, error bars represent 95% confidence interval.
