# Supplementary material for: Corrupted coordination of epigenetic modifications leads to diverging chromatin states and transcriptional heterogeneity in CLL
Source: Nat Commun. 2019 Apr 23;10:1874. doi: 10.1038/s41467-019-09645-5 (PMC6478836; doi:10.1038/s41467-019-09645-5)
Supplement: Supplementary file 2 — Description of Additional Supplementary Files [file 41467_2019_9645_MOESM2_ESM.pdf]

Description of Additional Supplementary Information

File Name: Supplementary Data 1.
Description: CLL and normal B cell samples used in this study from the Blueprint Initiative.

File Name: Supplementary Data 2.
Description: Transcription factor de novo motif enrichment between CLL and germinal center B cells at super-enhancers.

File Name: Supplementary Data 3.
Description: Transcription factor de novo motif enrichment between CLL and naïve B cells at super-enhancers. Related to Figure 1e.

File Name: Supplementary Data 4.
Description: Genomic coordinates of regions targeted with bisulfite sequencing capture assay.

File Name: Supplementary Data 5.
Description: Standard performance metrics for normal B and CLL patient samples profiled with targeted bisulfite sequencing capture assay.

File Name: Supplementary Data 6.
Description: Differentially methylated regions (DMRs; absolute change in DNAme > 0.3 and Fisher's exact test FDR < 0.05; see Methods) between CLL (both IGHV mutated and unmutated) and normal B samples.

File Name: Supplementary Data 7.
Description: Differentially methylated regions (DMRs; absolute change in DNAme > 0.3 and Fisher's exact test FDR < 0.05; see Methods) between IGHV mutated CLL and normal B samples.

File Name: Supplementary Data 8.
Description: Differentially methylated regions (DMRs; absolute change in DNAme > 0.3 and Fisher's exact test FDR < 0.05; see Methods) between IGHV unmutated CLL and normal B samples.

File Name: Supplementary Data 9.
Description: Gene sets and gene ontology enrichment for CLL hypomethylated super-enhancers. Related to Supplementary Figure 2g.

File Name: Supplementary Data 10.
Description: Gene sets (CGP) enriched in closest genes (average distance of 496 bp) to genomic segments that gain H3K27ac from normal B cells to CLL (hypergeometric test BH-FDR < 0.05). Related to Supplementary Figure 4e.

File Name: Supplementary Data 11.
Description: Primers and adapters used in MscRRBS library construction.

File Name: Supplementary Data 12.
Description: List of cell barcodes used in MscRRBS library construction.
