# Supplementary material for: Corrupted coordination of epigenetic modifications leads to diverging chromatin states and transcriptional heterogeneity in CLL
Source: Nat Commun. 2019 Apr 23;10:1874. doi: 10.1038/s41467-019-09645-5 (PMC6478836; doi:10.1038/s41467-019-09645-5)
Supplement: Supplementary file 15 — Reporting Summary [file 41467_2019_9645_MOESM15_ESM.pdf]

nature research

Corresponding author(s): Dan Landau

Last updated by author(s): Feb 26, 2019

Reporting Summary

Nature Research wishes to improve the reproducibility of the work that we publish. This form provides structure for consistency and transparency in reporting. For further information on Nature Research policies, see Authors & Referees and the Editorial Policy Checklist.

Statistics

For all statistical analyses, confirm that the following items are present in the figure legend, table legend, main text, or Methods section.

n/a	Confirmed	
☐	☒	The exact sample size (*n*) for each experimental group/condition, given as a discrete number and unit of measurement
☐	☒	A statement on whether measurements were taken from distinct samples or whether the same sample was measured repeatedly
☐	☒	The statistical test(s) used AND whether they are one- or two-sided<br>*Only common tests should be described solely by name; describe more complex techniques in the Methods section.*
☐	☒	A description of all covariates tested
☐	☒	A description of any assumptions or corrections, such as tests of normality and adjustment for multiple comparisons
☐	☒	A full description of the statistical parameters including central tendency (e.g. means) or other basic estimates (e.g. regression coefficient) AND variation (e.g. standard deviation) or associated estimates of uncertainty (e.g. confidence intervals)
☐	☒	For null hypothesis testing, the test statistic (e.g. *F*, *t*, *r*) with confidence intervals, effect sizes, degrees of freedom and *P* value noted<br>*Give P values as exact values whenever suitable.*
☒	☐	For Bayesian analysis, information on the choice of priors and Markov chain Monte Carlo settings
☒	☐	For hierarchical and complex designs, identification of the appropriate level for tests and full reporting of outcomes
☐	☒	Estimates of effect sizes (e.g. Cohen's *d*, Pearson's *r*), indicating how they were calculated

Our web collection on statistics for biologists contains articles on many of the points above.

Software and code

Policy information about availability of computer code

Data collection	No software was used for data collection.
Data analysis	BWA v0.7.17; Macs2 (v2.0.10); Homer; DESeq2; STAR (v2.5.2a); MAQ version 0.6.6; bsMap v2.9; Picard; MOABS; IGV; methylKit package; BEDTools v2.25.0; GREAT version 3.0.0; ABSOLUTE; Bismark (v.0.14.5); bowtie2-2.2.8; SCENIC analytical toolkit; EpicSeg; scikit-learn Python library (sklearn.mixture.BayesianGaussianMixture v0.19); Python 2.7.13; R version 3.4.2

For manuscripts utilizing custom algorithms or software that are central to the research but not yet described in published literature, software must be made available to editors/reviewers. We strongly encourage code deposition in a community repository (e.g. GitHub). See the Nature Research guidelines for submitting code & software for further information.

Data

Policy information about availability of data

All manuscripts must include a data availability statement. This statement should provide the following information, where applicable:

- Accession codes, unique identifiers, or web links for publicly available datasets
- A list of figures that have associated raw data
- A description of any restrictions on data availability

ChIP-seq, RNA-seq, and DNAme datasets have been deposited to the NCBI Gene Expression Omnibus (GEO) under accession number GSE119103. MscRRBS and single-cell Smart-seq2 datasets have been deposited to the NCBI GEO under accession number GSE109085. The dbGaP accession number for the whole-exome sequencing data reported in this paper is phs000435.v2.p1. H3K27me3 ChIP-seq data for primary human tonsillar naive B cells and tonsillar germinal center B cells were downloaded from NCBI GEO under accession number GSE45982. Previously published CLL and normal B cell ChIP-seq and RNA-seq datasets were downloaded from the Blueprint DCC portal under accession number EGAC00001000135.

Field-specific reporting

Please select the one below that is the best fit for your research. If you are not sure, read the appropriate sections before making your selection.

☒ Life sciences ☐ Behavioural & social sciences ☐ Ecological, evolutionary & environmental sciences

For a reference copy of the document with all sections, see nature.com/documents/nr-reporting-summary-flat.pdf

Life sciences study design

All studies must disclose on these points even when the disclosure is negative.

Sample size	We sequenced 12 and 20 B cell healthy donors and CLL patients, respectively, giving us enough statistical power to detect differences between samples in all the analyses reported in this study. In addition, we sequenced 384 single cells in total from 1 and 2 B cell healthy donors and CLL patients, respectively, enabling us to detect statistical significant differences at the single cell level (e.g., single-cell mutual information analysis and single-cell transcriptional entropy analysis).
Data exclusions	No data were excluded from the study.
Replication	We generated genome-wide maps of histone marks with non-overlapping regulatory functions (H3K4me3, H3K27ac, and H3K27me3) and transcriptome sequencing (bulk RNA-seq) in a cohort of 20 primary IGHV mutated and unmutated CLL (corresponding to the major known disease subtypes; n = 14 and n = 6, respectively), as well as 12 healthy B lymphocytes samples. In addition, we performed joint single-cell DNAme sequencing and whole transcriptome sequencing on additional normal B and CLL samples (n = 96 cells [1 sample], n = 288 cells [2 samples]). All attempts at replication were successful.
Randomization	Randomization is not applicable as no experimental groups were used in our study.
Blinding	Blinding is not applicable as no experimental groups were used in our study.

Reporting for specific materials, systems and methods

We require information from authors about some types of materials, experimental systems and methods used in many studies. Here, indicate whether each material, system or method listed is relevant to your study. If you are not sure if a list item applies to your research, read the appropriate section before selecting a response.

Materials & experimental systems

n/a	Involved in the study	
☐	☒	Antibodies
☒	☐	Eukaryotic cell lines
☒	☐	Palaeontology
☒	☐	Animals and other organisms
☐	☒	Human research participants
☒	☐	Clinical data

Methods

n/a	Involved in the study	
☐	☒	ChIP-seq
☒	☐	Flow cytometry
☒	☐	MRI-based neuroimaging

Antibodies

Antibodies used	Purified CD19+ naive B cells (CD19+CD23+CD27−IgD+) and germinal center memory B cells (CD19+CD23+CD27+IgD-) were sorted using PE/Cy7 anti-human CD27 Antibody (1:5 dilution; clone O323, Bio Legend) and FITC Mouse anti-human IgD (1:5 dilution; clone IA6-2, BD Pharmingen) antibodies with a FACSAria II instrument (Becton Dickinson, Franklin Lakes, NJ). Tonsillar CD20+ cells were sorted as CD19+CD20+CD38++.<br>Antibodies used for ChIP include anti-H3K4me3 (1 mg for 50 mg of chromatin; 9751S Cell Signaling, Danvers, MA), anti-H3K27ac (2 mg for 25 mg of chromatin; ab4729 Abcam, Cambridge, United Kingdom), anti-H3K27me3 (2 mg for 25 mg of chromatin; 07–449 Millipore, Burlington, MA).
Validation	All antibodies used were validated for their use in FACS or ChIP-seq experiments with human samples, as shown on the website provided by the respective companies.

Human research participants

Policy information about studies involving human research participants

Population characteristics	Relevant information on human research participants is Supplementary Figure 1b.
Recruitment	The diagnosis of CLL according to World Health Organization (WHO) criteria was confirmed in all cases by flow cytometry, or by

Recruitment	lymph node or bone marrow biopsy. Informed consent on DF/HCC, MSKCC and WCM IRB-approved protocols for genomic sequencing of patients' samples was obtained prior to the initiation of sequencing studies. Blood samples were collected in EDTA blood collection tubes (BD Biosciences) from patients and healthy adult volunteers enrolled on clinical research protocols at the Dana-Faber/Harvard Cancer Center (DF/HCC), Memorial Sloan Kettering Cancer Center (MSKCC), and NewYork-Presbyterian/ Weill Cornell Medical Center (NYP/WCMC).
Ethics oversight	The study was approved by the local ethics committee and by the Institutional Review Board (IRB) and conducted in accordance to the Declaration of Helsinki protocol. We note that the IRB does not permit collection of demographic information of healthy donors.

Note that full information on the approval of the study protocol must also be provided in the manuscript.

ChIP-seq

Data deposition

☒ Confirm that both raw and final processed data have been deposited in a public database such as GEO.

☒ Confirm that you have deposited or provided access to graph files (e.g. BED files) for the called peaks.

Data access links
May remain private before publication.

https://www.ncbi.nlm.nih.gov/geo/query/acc.cgi?acc=GSE119103

Files in database submission

GSM3358043 cb_1_rnaseq
GSM3358044 cb_2_rnaseq
GSM3358045 cll_10126_rnaseq
GSM3358046 cll_151_rnaseq
GSM3358047 cll_175_rnaseq
GSM3358048 cll_189_rnaseq
GSM3358049 cll_20506_rnaseq
GSM3358050 cll_20788_rnaseq
GSM3358051 cll_20792_rnaseq
GSM3358052 cll_20796_rnaseq
GSM3358053 cll_21626_rnaseq
GSM3358054 cll_242_rnaseq
GSM3358055 cll_248_rnaseq
GSM3358056 cll_253_rnaseq
GSM3358057 cll_32545_rnaseq
GSM3358058 cll_4605_rnaseq
GSM3358059 cll_4627_rnaseq
GSM3358060 cll_51_rnaseq
GSM3358061 cll_5268_rnaseq
GSM3358062 cll_67_rnaseq
GSM3358063 cll_73_rnaseq
GSM3358064 cll_75_rnaseq
GSM3358065 cll_95_rnaseq
GSM3358066 cll_97_rnaseq
GSM3358067 nb_1_rnaseq
GSM3358068 nb_2_rnaseq
GSM3358070 cd20_01835_h3k4me3_bam
GSM3358073 cd20_167_h3k27ac_bam
GSM3358074 cd20_167_h3k27me3_bam
GSM3358078 cll_175_h3k27ac_bam
GSM3358079 cll_175_h3k27me3_bam
GSM3358080 cll_175_h3k4me3_bam
GSM3358081 cll_175_wce_bam
GSM3358082 cll_189_h3k27ac_bam
GSM3358083 cll_189_h3k27me3_bam
GSM3358085 cll_189_h3k4me3_bam
GSM3358086 cll_189_wce_bam
GSM3358087 cll_20788t_h3k27ac_bam
GSM3358088 cll_20788t_h3k27me3_bam
GSM3358089 cll_20792t_h3k27ac_bam
GSM3358090 cll_20792t_h3k27me3_bam
GSM3358091 cll_21626t_h3k27ac_bam
GSM3358092 cll_21626t_h3k27me3_bam
GSM3358093 cll_22526t_h3k27ac_bam
GSM3358094 cll_22526t_h3k27me3_bam
GSM3358095 cll_242_h3k27ac_bam
GSM3358096 cll_242_h3k27me3_bam
GSM3358097 cll_242_h3k4me3_bam
GSM3358098 cll_242_wce_bam
GSM3358099 cll_248_h3k27ac_bam
GSM3358100 cll_248_h3k27me3_bam
GSM3358101 cll_248_h3k4me3_bam

GSM3358102 cll_248_wce_bam
GSM3358103 cll_253_h3k27ac_bam
GSM3358104 cll_253_h3k27me3_bam
GSM3358106 cll_253_h3k4me3_bam
GSM3358107 cll_253_wce_bam
GSM3358108 cll_32545t_h3k27ac_bam
GSM3358109 cll_32545t_h3k27me3_bam
GSM3358110 cll_4605t_h3k27ac_bam
GSM3358111 cll_4605t_h3k27me3_bam
GSM3358112 cll_51_h3k27ac_bam
GSM3358113 cll_51_h3k27me3_bam
GSM3358114 cll_51_h3k4me3_bam
GSM3358115 cll_51_wce_bam
GSM3358116 cll_5268t_h3k27ac_bam
GSM3358117 cll_5268t_h3k27me3_bam
GSM3358118 cll_7528t_h3k27ac_bam
GSM3358119 cll_7528t_h3k27me3_bam

Genome browser session
(e.g. UCSC)

No longer applicable.

Methodology

Replicates

20 primary IGHV mutated and unmutated CLL (corresponding to the major known disease subtypes; n = 14 and n = 6, respectively), as well as 12 healthy B lymphocytes samples.

Sequencing depth

125 bp paired-end mode. An average of 75 million paired reads was generated per sample

Antibodies

Antibodies used for ChIP include anti-H3K4me3 (1 mg for 50 mg of chromatin; 9751S Cell Signaling, Danvers, MA), anti-H3K27ac (2 mg for 25 mg of chromatin; ab4729 Abcam, Cambridge, United Kingdom), anti-H3K27me3 (2 mg for 25 mg of chromatin; 07–449 Millipore, Burlington, MA).

Peak calling parameters

Peaks were identified with Macs2 (v2.0.10) with a q-value threshold of 0.01, according to the ENCODE Histone ChIP-seq Data Standards and Processing Pipeline (https://www.encodeproject.org/chip-seq/histone/).

Data quality

Deeptools plotFingerprint v2 was used to assess ChIP-seq signal enrichment over background signal.
As ChIP-seq experiments are prone to technical variation, we further demonstrated the reproducibility of our ChIP-seq datasets in CLL by analyzing additional CLL and normal B cell samples from the Blueprint Initiative (Supplementary Table 1), showing high pairwise correlations across our cohort and the Blueprint initiative samples (Supplementary Figure 1e).

Software

ChIP-seq data were processed according to the ENCODE Histone ChIP-seq Data Standards and Processing Pipeline (https://www.encodeproject.org/chip-seq/histone/). Raw reads were mapped to the human genome GRCh37 assembly using Burrows-Wheeler Aligner (BWA v0.7.17). Duplicate reads were removed using Picard (https://broadinstitute.github.io/picard/) and bigwig files were created for visualization. Peaks were identified with Macs2 (v2.0.10) with a q-value threshold of 0.01. Peaks overlapping with Satellite repeat regions and Encode Blacklist were discarded.
